# Analysis and Computational Dissection of Molecular Signature Multiplicity

**DOI:** 10.1371/journal.pcbi.1000790

**Published:** 2010-05-20

**Authors:** Alexander Statnikov, Constantin F. Aliferis

**Affiliations:** 1 Center for Health Informatics and Bioinformatics, New York University School of Medicine, New York, New York, United States of America; 2 Department of Medicine, New York University School of Medicine, New York, New York, United States of America; 3 Department of Pathology, New York University School of Medicine, New York, New York, United States of America; 4 Department of Biomedical Informatics, Vanderbilt University, Nashville, Tennessee, United States of America; 5 Department of Biostatistics, Vanderbilt University, Nashville, Tennessee, United States of America; Accelrys, United States of America

## Abstract

Molecular signatures are computational or mathematical models created to diagnose disease and other phenotypes and to predict clinical outcomes and response to treatment. It is widely recognized that molecular signatures constitute one of the most important translational and basic science developments enabled by recent high-throughput molecular assays. A perplexing phenomenon that characterizes high-throughput data analysis is the ubiquitous multiplicity of molecular signatures. Multiplicity is a special form of data analysis instability in which different analysis methods used on the same data, or different samples from the same population lead to different but apparently maximally predictive signatures. This phenomenon has far-reaching implications for biological discovery and development of next generation patient diagnostics and personalized treatments. Currently the causes and interpretation of signature multiplicity are unknown, and several, often contradictory, conjectures have been made to explain it. We present a formal characterization of signature multiplicity and a new efficient algorithm that offers theoretical guarantees for extracting the set of maximally predictive and non-redundant signatures independent of distribution. The new algorithm identifies exactly the set of optimal signatures in controlled experiments and yields signatures with significantly better predictivity and reproducibility than previous algorithms in human microarray gene expression datasets. Our results shed light on the causes of signature multiplicity, provide computational tools for studying it empirically and introduce a framework for in silico bioequivalence of this important new class of diagnostic and personalized medicine modalities.

## Author Summary

One of the promises of personalized medicine is to use molecular information to better diagnose, manage, and treat disease. This promise is enabled through the use of molecular signatures that are computational models to predict a phenotype of interest from high-throughput assay data. Many molecular signatures have been developed to date, and some passed regulatory approval and are currently used in clinical practice. However, researchers have noted that it is possible to develop many different and equivalently accurate molecular signatures for the same phenotype and population. This phenomenon of signature multiplicity has far-reaching implications for biological discovery and development of next generation patient diagnostics and personalized treatments. Currently the causes and interpretation of signature multiplicity are unknown, and several, often contradictory, conjectures have been made to explain it. Our results shed light on the causes of signature multiplicity and provide a method for extracting all equivalently accurate signatures from high-throughput data.

## Introduction

A *molecular signature* is a computational or mathematical model that predicts a phenotype of interest (e.g., diagnosis or outcome of treatment in human patients or biological models of disease) from microarray gene expression or other high-throughput assay data inputs [1], [2]. *Multiplicity* is a special form of data analysis instability in which different analysis methods used on the same data, or different samples from the same population lead to *different but apparently maximally predictive* signatures [3], [4]. This phenomenon has far-reaching implications for biological discovery and development of next generation patient diagnostics and personalized treatments. Multiplicity in the *best case* implies that generation of biological hypotheses (e.g., discovery of potential drug targets) is very hard even when signatures are maximally predictive of the phenotype since thousands of completely different signatures are equally consistent with the data. In the *worst case* this phenomenon entails that the produced signatures are not statistically generalizable to new cases, and thus not reliable enough for translation to clinical practice.

Some authors motivated by classical statistical considerations, attribute signature multiplicity solely to the small sample size of typical microarray gene expression studies [5] and have conjectured that it leads to non-reproducible predictivity when the signatures are applied in independent data [6]. Related to the above it has been suggested that building reproducible signatures requires thousands of observations [7]. Other authors have proposed that the phenomenon of signature multiplicity is a byproduct of the complex regulatory connectivity of the underlying biological system leading to existence of highly predictively redundant biomarker sets [8]. The specifics of what types of connectivity or regulatory relationships may lead to multiplicity have not been concretely identified however. Another possible explanation of signature multiplicity is implicit in previously described artifacts of data pre-processing. For example, normalization may inflate correlations between genes, making some of them interchangeable for prediction of the phenotype [9]–[11].

Critical to the ability to study the phenomenon empirically is the availability of computational methods capable of extracting multiple signatures from the data. Several methods have been introduced with this intent. The available methods encompass four algorithm families. The first family is *resampling-based signature extraction*. It operates by repeated application of a signature extraction algorithm to resampled data (e.g., via bootstrapping) [6], [12], [13]. This family of methods is based on the assumption that multiplicity is strictly a small sample phenomenon. The second family is *iterative removal*, that is repeating signature extraction after removing from the data all genes that have been found in the previously discovered molecular signatures [14]. This approach is agnostic as to what causes multiplicity and is heuristic since it does not propose a theory of causes of multiplicity. The third family is *stochastic gene selection* techniques [15], [16]. The underlying premise of the method of [15] is that in a specific class of distributions every maximally predictive and non-redundant signature will be output by a randomized algorithm with non-zero probability (thus all such signatures will be output when the algorithm is applied an infinite number of times). Similarly, the method of [16] will output all signatures discoverable by a genetic algorithm when it is allowed to evolve an infinite number of populations. The fourth family is *brute force exhaustive search* [17]. This approach is agnostic as to what causes multiplicity, and requires time that is exponential to the total number of genes, thus it is computationally infeasible for signatures with more than 2–3 genes (as almost all maximally predictive signatures are in practice).

The above methods, useful first attempts as they may be, are either heuristic or computationally intractable, are based on currently unvalidated conjectures about what causes multiplicity, and output incomplete sets of signatures with currently unknown generalizability. The practical benefits of an algorithm that could systematically extract the set of truly maximally predictive and non-redundant signatures include: (i) a deeper understanding of the signature multiplicity phenomenon and how it affects reproducibility of signatures; (ii) improving discovery of the underlying biological mechanisms by not missing genes that are implicated mechanistically in the disease processes; and (iii) catalyzing regulatory approval by establishing *in-silico equivalence* to previously validated signatures in a manner similar to bioequivalence of drugs.

To achieve these goals we provide a theoretical framework based on Markov boundary induction that enables probabilistic modeling of multiple signatures and formally connects it with the causal graph (i.e., pathways) of the data generating process [18]–[21] even when these pathways are not known a priori. We introduce a provably correct algorithm (termed TIE*) that outputs the set of maximally predictive and non-redundant signatures independent of the data distribution. We present experiments with real and resimulated microarray gene expression datasets as well as with artificial simulated data that verify the theoretical properties of TIE* and showcase its advantages over previous methods in practical settings. In particular, it is shown that TIE* having excellent sample and computational efficiency not only extracts many more maximally predictive and non-redundant signatures than all previous methods, but also that TIE* signatures are reproducible in independent datasets whereas signatures produced by previous methods are often not reproducible or have lower predictivity. The theoretical and experimental results obtained in the present study also suggest that some of the previous hypotheses about the causes and implications of signature multiplicity have to be radically reevaluated.

## Materials and Methods

### On analysis of signatures

To simplify analysis, and without loss of generality, instead of considering all possible signatures derivable from a given dataset (via a potentially infinite variety of classifier algorithms) we only consider the signatures that have maximal predictivity for the phenotypic response variable *relative to the genes (variables) contained in each signature*. In other words, we exclude from consideration signatures that do not utilize all predictive information about the phenotypic response variable contained in their genes. This allows us to study signature classes by reference only to the genes contained in each class. Specifically, for a gene set **X** there can be an infinite number of classifiers that achieve maximal predictivity for the phenotype relative to the information contained in **X**. Thus, when we say “signature **X**” we refer to one of these predictively equivalent classifiers. This reduction is justified, for example, whenever the classifiers used can learn the minimum error decision function given sufficient sample (for a given set of genes **X**, the minimal error decision function minimizes the error of predicting the phenotypic variable *T* given **X** over all possible decision functions). Most practical classifiers employed in this domain as well as classifiers used in our experiments (SVMs) satisfy the above requirement either on theoretical [22], [23] and/or empirical grounds [24].

Given the above reduction of signatures to equivalence classes, the focus of this work is in extracting signatures that satisfy two desirable optimality properties: (a) *maximally predictive of the phenotype* (informally this means that they can form the inputs to a predictor of the phenotype which for the given dataset and population cannot be improved by any other classifier-gene subset combination), and at the same time (b) *do not contain predictively redundant genes* (i.e., genes that can be removed from the signature without adversely affecting the signature predictivity). Every suboptimal signature (i.e., one that does not satisfy these two properties) can be discarded from consideration when studying multiplicity.

### Markov boundary characterization of signature multiplicity

As is proved in Text S1, two signatures **X** and **Y** of the phenotypic response variable *T* are maximally predictive and non-redundant if and only if **X** and **Y** are Markov boundaries of *T*. A Markov boundary **M** of *T* is a set of variables that (i) renders all other variables outside **M** and *T* independent of *T* conditioned on **M** (i.e., **M** is a Markov blanket of *T*) and (ii) no proper subset of **M** is a Markov blanket of *T* [18]. This definition also implies causal interpretability of **M** under distributional assumptions [18]–[21]. It was shown previously that the so-called *intersection property* of probability distributions is a sufficient condition for uniqueness of Markov boundaries [18], therefore it is also a sufficient condition for uniqueness of optimal molecular signatures. However, the extent to which signature multiplicity is present in distributions that violate the intersection property is not known.

**Figure 1:**
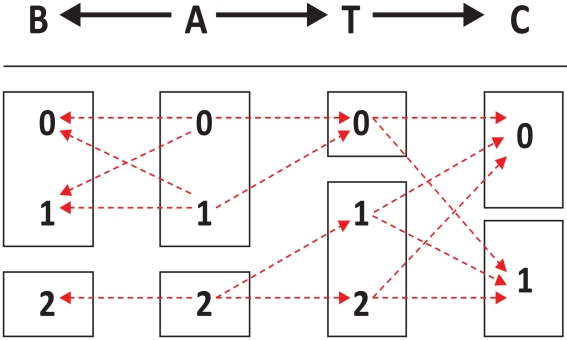
The figure describes a class of Bayesian networks that share the same pathway structure (with 3 gene variables *A*, *B*, *C* and a phenotypic response variable *T*) and their joint probability distribution obeys the constraints shown below the structure. Red dashed arrows denote nonzero conditional probabilities of each variable given its direct causes, and the absence of red dashed arrows denotes that these conditional probabilities are zero. For example, P(*T* = 0 | *A* = 1)≠0 while P(*T* = 0 | *A* = 2)  = 0. Genes *A*, *B* and phenotypic response variable *T* take 3 values {0, 1, 2}, while gene *C* takes two values {0, 1}.

Figure 1 shows by means of an illustrative example implications of signature multiplicity. It describes a class of Bayesian networks that share the same pathway structure (with three gene variables *A*, *B*, *C* and a phenotypic response variable *T*) and constraints on their joint probability distributions. Each member of this class is derived by parameterizing the joint probability distribution subject to the constraints. An example of a parameterized Bayesian network is provided in Figure S1. The following hold in all Bayesian networks that belong to this example class:

- There exist two maximally predictive and non-redundant signatures (Markov boundaries) of *T*: {*A*, *C*} and {*B*, *C*}. Furthermore, {*A*, *C*} and {*B*, *C*} remain maximally predictive and non-redundant signatures even in infinite samples from that distribution (i.e., *multiplicity does not vanish in the large sample*).
- The pathway structure has very low connectivity (e.g., maximum in-degree  = 1 and maximum out-degree  = 2) (i.e., *multiplicity does not require very dense connectivity*).
- *A* and *B* are not deterministically related, yet they convey individually the same information about *T* (i.e., *multiplicity does not require deterministic equivalence or extreme collinearity*).
- If an algorithm selects only one maximally predictive and non-redundant signature (e.g., {*B*, *C*}), then there is danger of missing biologically important (causative) genes (i.e., *A*) and focusing instead on confounded genes (i.e., *B*) (i.e., *only some of the predictively equivalent signatures have local causal interpretation*).
- The union of all maximally predictive and non-redundant signatures includes all genes located in the *local* pathway around *T*, i.e., *A* and *C* (we define *local pathway* as genes directly upstream or downstream of the response variable *T*.).
- In this example, the intersection of all maximally predictive and non-redundant signatures contains only genes in the *local* pathway around *T* (i.e., *C*).

The above example is concerned with the large sample case. In practice, one deals with small samples where statistical inferences have to be made about large sample predictivity and redundancy. This creates an additional source of error and concomitant multiplicity. An example of this is given in Text S2.

### TIE* algorithm for identification of the set of maximally predictive and non-redundant signatures

**Figure 2:**
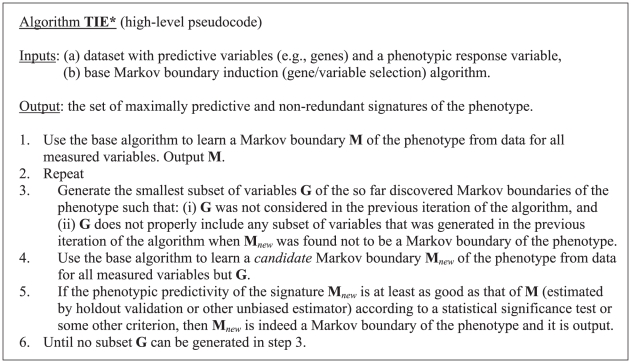
High-level pseudocode of the TIE* algorithm. Non-redundancy is not explicitly checked during the operation of TIE* but is a required property of the base Markov boundary algorithm. Details are provided in Text S3.

Figure 2 presents the high-level operation of the TIE* algorithm that uses Markov boundary induction to identify the set of maximally predictive and non-redundant signatures. Text S3 provides an example trace, proof of correctness, and implementation details of the algorithm. In step 1, TIE* uses a base Markov boundary induction algorithm that identifies a single molecular signature **M** of the phenotype with maximal predictivity and no redundancy (Markov boundary). The same base algorithm is applied repeatedly to versions of the original dataset in which some subset of variables **G** has been removed (step 4). If a new signature **M** *_new_* has the same predictivity for the phenotype as **M**, then it is a Markov boundary and it is output (step 5). Steps 3–5 are repeated until no subset **G** can be generated in step 3.

The base Markov boundary induction algorithm must be suitable for the distribution at hand. Thus, TIE* is a generative algorithm that is instantiated differently for different distributions. In the experiments reported in this paper, we use as the base algorithm HITON-PC (Figure S2), which is an instance of a very broad class of Markov boundary inducers termed *Generalized Local Learning* [25], [26]. This choice of the base algorithm is motivated by its empirical performance in microarray gene expression and other high-throughput data as well as its theoretical properties [25]–[28].

TIE* is guaranteed to be correct in the large sample under its stated assumptions. In the small sample some signatures that are not maximally predictive and/or redundant will be statistically indistinguishable from the maximally predictive and non-redundant ones. This indistinguishability occurs at two different levels: one is estimation of predictivity and testing for statistical significance of differences in predictivity among signatures. The second level is the performance of tests of conditional independence (or functional equivalents such as Bayesian scoring) with small samples inside the base algorithm which incurs errors of type I and II. As the sample size grows, the algorithm will output only truly maximally predictive and non-redundant signatures.

We present several experiments testing the new algorithm and comparing it against 8 previously described multiple signature extraction methods. The methods comprise of four resampling-based algorithms, one iterative removal method, and three stochastic gene selection methods (details in Text S4). Brute force exhaustive search and genetic algorithms were not applied due to their computational intractability.

Before applying TIE* to real data, we test its behavior in controlled (i.e., simulated and resimulated data) experiments where generative models are known and in the case of simulated data all maximally predictive and non-redundant signatures are known as well (details about data generation are provided in Text S5). This allows us to test whether the algorithm behaves according to theoretical expectations, whether it is robust to moderate sample sizes, and whether it is sensitive to high dimensionality. This also provides clues about the behavior of TIE* and the baseline comparison algorithms in our experiments with real human microarray data.

### Reproducibility testing protocol, human microarray gene expression datasets, and empirical criterion for assessing maximal predictivity of signatures

To test reproducibility of molecular signatures, we adopt an experimental design where one microarray dataset (“*discovery dataset*”) is used for identification of signatures and estimation of their predictivity by holdout validation [29], and another independent dataset (“*validation dataset*”) originating either from a *different laboratory* or from a *different microarray platform* is used for validation of predictivity of the signatures. No overlap of subjects between discovery and validation dataset analyses occurs in this design. The criteria for dataset admissibility and exact protocol for quality assurance and processing of pairs of datasets is described in Text S6. In total, 6 pairs of gene expression microarray datasets covering both human cancer diagnosis and outcome prediction were used (listed in Table S1).

Operationally we define maximal predictivity for each dataset as follows: we apply all tested methods for extraction of multiple signatures to a dataset; then for each method we compute average predictivity of the phenotype (over all identified signatures by this method) measured by area under ROC curve (AUC); finally we compute the maximum value of the above average predictivity estimates and refer to it as “maximal predictivity”.

Statistical comparisons of predictivity between methods in the same dataset are accomplished by Wilcoxon rank sum test with α = 0.05 [30]. This is a two-sided test of the null hypothesis that two samples come from distributions with equal medians. When we use this test, the first sample contains AUC estimates of all signatures identified by one multiple signature extraction method; and the second sample contains AUC estimates of all signatures identified by another method.

## Results

### Experiments with artificial simulated data

Tables S2 and S3 present the results of experiments with TIE* and baseline comparison algorithms. The following are observed: (i) TIE* perfectly identifies all 72 maximally predictive and non-redundant signatures that exist in the distribution using datasets with either 30 or 1,000 variables; (ii) iterative removal identifies only 1 signature because all other signatures have a common variable and thus cannot be detected by this method; (iii) KIAMB fails to identify any optimal signature due to its sample inefficiency, and because of the same reason its signatures have poor classification performance; (iv) resampling-based methods either miss many optimal signatures and/or output many redundant variables in the signatures.

### Experiments with resimulated gene expression microarray data

We applied TIE* to resimulated gene expression data with sample sizes: 300, 450,…, 1500, 2250, 3000,…, 30000. A signature is operationally considered as *non-reducible* if it is not properly included in any other signature output by this method (i.e., it is a proxy of having no redundant genes). For example, if a method outputs 3 signatures with the following genes: {*A*, *B*, *C*}, {*A*, *B*, *X*}, and {*A*, *B*}, only signature {*A*, *B*} is non-reducible. The number of maximally predictive signatures (as confirmed in independent data by holdout validation) and the number of maximally predictive and non-reducible signatures output by the algorithm for each sample size in resimulated data is shown in Figure S3. As sample size increases, the number of output maximally predictive signatures drops but then remains constant in the range 160–644 (or 53–279 for non-reducible signatures) for datasets with ≥4,500 samples. *This is consistent with the existence of at least two sources of multiplicit*y: one is small sample size and the other is multiplicity intrinsic to the nature of gene-gene and gene-phenotype relations. As sample size grows, the first source vanishes and only the second one remains. Since the resimulated data distribution closely mimics the real-life distribution (Text S5), this experiment supports the hypothesized existence of multiple maximally predictive and non-redundant signatures in very large samples (>10,000) contrary to the theoretical model of [5].

### Experiments with real human data showing that signatures produced by TIE* have maximal predictivity in independent validation datasets

Table S4 shows that TIE* achieves maximal predictivity in 5 out of 6 human microarray validation datasets. Non-TIE* methods achieve maximal predictivity in 0 to 2 datasets depending on the method. In the dataset where TIE* has predictivity that is statistically distinguishable from the empirical maximal one (*Lung Cancer Subtype Classification*), the magnitude of this difference is <0.009 AUC on average over all discovered signatures; thus this particular deviation from maximal predictivity may be considered negligible for most practical purposes.

**Figure 3:**
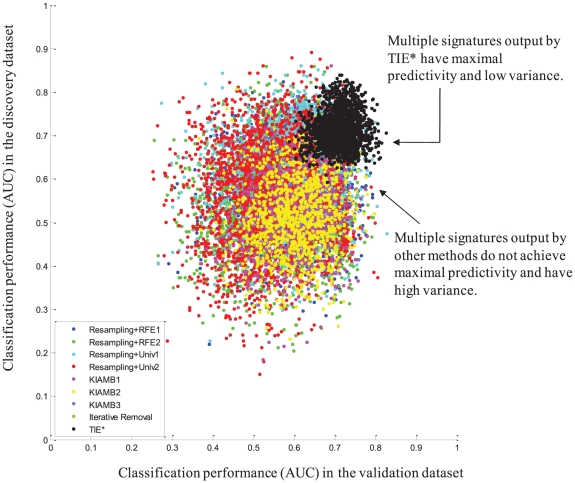
Plot of classification performance (AUC) in the validation dataset versus classification performance in the discovery dataset for each signature output by each method for the *Leukemia 5 yr. Prognosis* task. Each dot in the graph corresponds to a signature (SVM computational model of the phenotype).

A detailed example of application of multiple signature extraction methods to the *Leukemia 5 Yr. Prognosis* task is provided in Figure 3. The figure shows predictivity estimated in the discovery dataset (using an unbiased error estimator and protocol) against predictivity verified in the validation dataset for each signature. As can be seen, TIE* signatures have superior predictivity and lower variance compared to the signatures output by other methods. Similar behavior can be observed in other tasks as well.

### Experiments with real human data showing that signatures produced by TIE* are statistically reproducible whereas signatures from other methods are often overfitted

**Figure 4:**
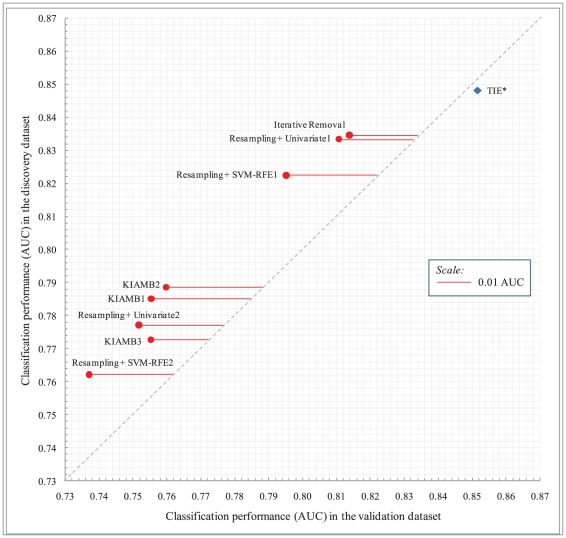
Plot of classification performance (AUC) in the validation dataset versus classification performance in the discovery dataset averaged over 6 pairs of datasets. Axes are magnified for better visualization.

Figure 4 plots predictivity estimated in the discovery dataset (using an unbiased error estimator and protocol) against predictivity verified in the validation dataset for all methods *averaged over all datasets and all discovered signatures*. Recall that validation datasets originate from different laboratories and/or using different microarray platforms than discovery datasets. The horizontal distance of each method to the diagonal measures the magnitude of overfitting defined as the difference (ε_1_-ε_2_), where ε_1_  =  expected performance in the validation data obtained by holdout validation in the discovery dataset, and ε_2_  =  observed validation dataset performance. TIE* rests slightly right of the diagonal denoting no overfitting, or equivalently perfect statistical reproducibility on average. However all other methods exhibit varying degrees of non-reproducibility. Depending on method the average magnitude of overfitting varies from 0.02 to 0.03 AUC.

### Signatures produced by TIE* in human microarray data have many genes in common

Analysis of the signatures output by TIE* reveals that they share many genes in common. Table S5 shows the number of common genes in 50%, 60%, …, 100% of output signatures for each dataset. Genes differ in the percentage of signatures they participate in. A heuristic that genes that belong to a larger fraction of signatures are localized closer to the pathway(s) affecting and being affected by the phenotypic response variable may be useful in exploratory studies, however this does not hold in all distributions [31].

## Discussion

### Computational complexity of the multiple signature discovery problem

The properties of the data-generative process affect computational feasibility of the signature discovery. In the worst case, it is computationally infeasible to discover even one of all optimal signatures with all known sound algorithms (i.e., algorithms that under specific conditions provably guarantee to provide the desired output; for the purposes of the present paper, to find a signature that is optimal in the population). However, there exist several sound algorithms for extracting an optimal signature that run in low-order polynomial time in real high-throughput data (e.g., HITON-PC). Even if the computational cost of discovery of one signature was constant, the number of all optimal signatures can grow exponentially large in the number of genes measured (for an example see Text S7). Thus the computational cost of dissecting signature multiplicity ranges from low-order polynomial (tractable) to super-exponential (infeasible) depending on the distribution. The worst-case characteristics are a property of the distribution analyzed and not the algorithm employed. One can thus only hope that real-life high-throughput data distributions are not representative of the worst-case theoretical ones. In addition, algorithms are needed that exploit the structure of the generative process to discover multiple signatures efficiently when the distribution allows it. Our experiments support that real-life data does not behave as the worst-case expected theoretical scenarios because TIE* terminated within at most several hours in each of the 6 microarray datasets that contains more than 10,000 oligonucleotide probes (using a Matlab implementation on a workstation with a single Intel Xeon 2.4 GHz processor and 4Gb of RAM). One can postulate various reasons for the tractability such as: (a) that biological pathways are sufficiently sparse thus not allowing for an exponential number of optimal signatures; (b) that to the extent that multiplicity denotes biologically redundant function, there is an “economy” of such redundant mechanisms, and (c) that a very large number of optimal signatures requires constraints on the network topology that are inconsistent with the structure of many biologically functional pathways.

### Understanding causes of signature multiplicity

The results of the present study refute or suggest that modifications are needed to several widespread positions about causes of signature multiplicity. The example model pathway in Figure 1 demonstrates that signature reproducibility neither precludes multiplicity nor requires sample sizes with thousands of subjects. It also shows that multiplicity of signatures does not require dense connectivity of the underlying pathways. Similarly, it shows that noisy measurements or normalization are not necessary conditions for signature multiplicity. The resimulation experimental data suggest that networks modeled on real gene expression data can exhibit signature multiplicity even in large sample sizes and that in this type of data, multiplicity is produced by a combination of small sample size-related variance *and* intrinsic multiplicity in the underlying network. The results with real human microarray datasets show that multiple signatures output by TIE* are reproducible and maximally predictive even though they are derived from small sample, noisy, and heavily-processed data.

Our results are consistent with the hypothesis that signature multiplicity in real-life datasets is created by a combination of several factors that include the following: *First*, the intrinsic information redundancy (due to gene-gene and gene-phenotype relations) in the complex regulatory network of the underlying biological system. *Second*, the variability in the output of gene selection and classifier algorithms especially in small sample sizes. *Third*, the small sample statistical indistinguishability of signatures that have different large sample predictivity and/or redundancy characteristics (example is given in Text S2). *Fourth*, the presence of hidden/unobserved variables (example is given in Text S8). *Fifth*, correlated measurement noise components that introduce a bias in gene expression profiles (e.g., noise that is localized in regions of microarray chips) [32]. *Sixth*, RNA amplification techniques that systematically distort measurements of transcript ratios (e.g., double-round T7-based amplification protocol) [33]. *Seventh*, cellular aggregation and sampling from mixtures of distributions that affect inference of conditional independence relations that are needed to establish model equivalence according to our framework for multiplicity [34]. *Eighth*, normalization and other data pre-processing methods that artificially increase correlations among genes (e.g., multivariate normalization in microarrays) [9]–[11]. Finally *ninth*, the engineered redundancy in the assay technology platforms (e.g., multiple probes for the same gene). In datasets produced by dissimilar underlying biological mechanisms, assayed with different platforms and pre-processed and modeled with a variety of algorithms, the relative contributions of the above factors to multiplicity will vary. As a result, methods that rely on a specific cause of multiplicity or combination of causes will not output all maximally predictive and non-redundant signatures in all types of high-throughput data.

### Analysis of methods for multiple signature extraction

With regard to non-TIE* baseline comparison algorithms, we note that resampling-based methods that use bootstrap samples to extract signatures may stop producing multiple signatures in large sample sizes. This is expected because resampling methods are designed to address directly only the small sample multiplicity and not the intrinsic multiplicity which persists in large samples. Iterative removal, on the other hand, by its design always fails to identify all maximally predictive and non-redundant signatures that have genes in common. KIAMB among the baseline algorithms has the strongest theoretical motivation because it can be shown to discover all Markov boundaries for a subset of distributions. However, a major limitation of KIAMB is that it has sample size requirements that range from at least linear to exponential to the number of genes in a signature (depending on the test of independence employed). This makes the algorithm not only computationally inefficient but also prone to statistical errors in small sample sizes. This leads to its substantial observed overfitting in the experiments with real data and its inability to find the maximally predictive and non-redundant signatures in simulated data. KIAMB, being a randomized search algorithm, also guarantees to output all optimal signatures that satisfy its distributional requirements, but only after infinite number of runs. The method by design will discover the same signatures over and over again further compounding its computational inefficiency.

Dealing with molecular signature multiplicity using a Markov boundary framework and the TIE* algorithm does not require a particular combination of factors causing signature multiplicity in order to be able to discover all maximally predictive and non-redundant signatures. Because of efficient heuristics, TIE* can extract the signature set very quickly when the connectivity is locally sparse, and the number of true optimal signatures is low-order polynomial or smaller in the number of variables. A very important factor for the performance of TIE* is the choice of the base algorithm to discover non-redundant and maximally predictive signatures in the distribution at hand. Latest developments in Markov boundary discovery provide such tools for high-throughput data. One of the key advantages of these methods is their ability to implicitly control for false discovery rate [25].

### Future research

Our experiments used real data exclusively from human cancer gene expression microarrays because of pragmatic reasons: known identity of observed variables, number and size of datasets, and maturity of standardization protocols that allows for multiple independent dataset validation experiments. The methods introduced here are however directly applicable to any high-throughput data, and future research in this direction is warranted. As an example of applicability of TIE* to high-throughput data beyond gene expression microarrays, we applied the method to proteomics mass-spectrometry data where TIE* identified hundreds of signatures of ovarian cancer with AUC = 0.95−0.98 (details in Text S9).

## Supporting Information

Figure S1 Example pathway structure with 3 gene variables (*A, B, C*) and phenotypic response variable *T*.
(0.02 MB PDF)

Figure S2 HITON-PC algorithm.
(0.05 MB PDF)

Figure S3 Number of signatures output by TIE* as sample size grows.
(0.01 MB PDF)

Table S1 Gene expression microarray datasets used in this work.
(0.03 MB PDF)

Table S2 Results of experiments with artificial dataset with 30 variables.
(0.02 MB PDF)

Table S3 Results of experiments with artificial dataset with 1,000 variables.
(0.02 MB PDF)

Table S4 Results for the number of output signatures (total/unique/unique and non-reducible), number of genes in a signature, and classification performance in discovery and validation microarray datasets.
(0.06 MB PDF)

Table S5 Number of common genes in 50%, 60%, …, 100% of signatures discovered by TIE* algorithm for each dataset.
(0.04 MB PDF)

Text S1 Maximally predictive and non-redundant molecular signatures are precisely the Markov boundaries and vice-versa.
(0.07 MB PDF)

Text S2 An example of signature multiplicity due to small samples.
(0.08 MB PDF)

Text S3 Details about the TIE* algorithm.
(0.15 MB PDF)

Text S4 Previous algorithms for multiple signature identification used in experiments.
(0.08 MB PDF)

Text S5 Data generation details.
(0.13 MB PDF)

Text S6 Criteria for microarray gene expression dataset admissibility and protocol for quality assurance and processing.
(0.04 MB PDF)

Text S7 The number of maximally predictive and non-redundant signatures is worst-case exponential to the number of variables.
(0.08 MB PDF)

Text S8 An example of signature multiplicity due to hidden variables.
(0.02 MB PDF)

Text S9 Preliminary experiments with the TIE* algorithm in mass-spectrometry proteomics data.
(0.02 MB PDF)
