# Supplementary material for: Analysis and Computational Dissection of Molecular Signature Multiplicity
Source: PLoS Comput Biol. 2010 May 20;6(5):e1000790. doi: 10.1371/journal.pcbi.1000790 (PMC2873900; doi:10.1371/journal.pcbi.1000790)
Supplement: Figure S1 — Example pathway structure with 3 gene variables (A, B, C) and phenotypic response variable T. (0.02 MB PDF) [file pcbi.1000790.s001.pdf]

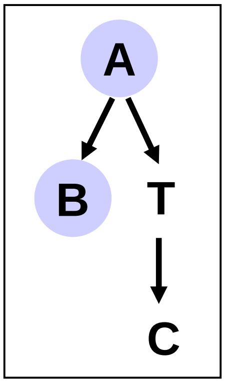

P(A)	
A = 0	0.5
A = 1	0.3
A = 2	0.2

P(T | A)	A = 0	A = 1	A = 2
T = 0	1	1	0
T = 1	0	0	0.3
T = 2	0	0	0.7

P(B | A)	A = 0	A = 1	A = 2
B = 0	0.2	0.9	0
B = 1	0.8	0.1	0
B = 2	0	0	1

P(C | T)	T = 0	T = 1	T = 2
C = 0	0.2	0.9	0.3
C = 1	0.8	0.1	0.7

Figure S1: Example pathway structure with 3 gene variables (A, B, C) and phenotypic response variable T. The structure is represented by a Bayesian network (defined as a set of variables **V**, a directed acyclic graph G with one-to-one correspondence of nodes to the variables in **V**, and a probability distribution P over **V** such that every variable W in **V** is independent of all non-descendants of W given the parents of W). The network parameterization is defined below the graph. All variables take values {0,1,2} except for C that takes values {0,1}. Genes A and B contain exactly the same information about T and are highlighted with the same color.
