# Supplementary material for: Analysis and Computational Dissection of Molecular Signature Multiplicity
Source: PLoS Comput Biol. 2010 May 20;6(5):e1000790. doi: 10.1371/journal.pcbi.1000790 (PMC2873900; doi:10.1371/journal.pcbi.1000790)
Supplement: Figure S2 — HITON-PC algorithm. (0.05 MB PDF) [file pcbi.1000790.s002.pdf]

Algorithm **HITON-PC** (without "symmetry correction")

Input: dataset with predictive variables (e.g., genes) **V**, including a phenotypic response variable *T*

Output: a Markov boundary **M** of *T*

1. Initialize **M** with an empty set
2. Initialize the set of eligible variables **E** ← **V** \ {*T*}
3. Sort in descending order the variables in **E** according to their pairwise association with response variable *T*
4. Remove from **E** all variables *X* with zero association with *T*, i.e. when $T \perp X$
5. Repeat
6. *X* ← first variable in **E**
7. Add *X* to **M** and remove it from **E**
8. If ∃ **Z** ⊆ **M** \ {*X*}, such that $T \perp X \mid \mathbf{Z}$, remove *X* from **M**
9. Until **E** is empty
10. For each $X \in$ **M**
11. If ∃ **Z** ⊆ **M** \ {*X*}, such that $T \perp X \mid \mathbf{Z}$, remove *X* from **M**
12. Output **M**

Figure S2: HITON-PC algorithm. More details about the algorithm and characterization of distributions where it can correctly identify a Markov boundary are given in [1,2].

References

1. Aliferis CF, Statnikov A, Tsamardinos I, Mani S, Koutsoukos XD (2010) Local Causal and Markov Blanket Induction for Causal Discovery and Feature Selection for Classification. Part II: Analysis and Extensions. Journal of Machine Learning Research 11: 235-284.

2. Aliferis CF, Statnikov A, Tsamardinos I, Mani S, Koutsoukos XD (2010) Local Causal and Markov Blanket Induction for Causal Discovery and Feature Selection for Classification. Part I: Algorithms and Empirical Evaluation. Journal of Machine Learning Research 11: 171-234.
