# Supplementary material for: Analysis and Computational Dissection of Molecular Signature Multiplicity
Source: PLoS Comput Biol. 2010 May 20;6(5):e1000790. doi: 10.1371/journal.pcbi.1000790 (PMC2873900; doi:10.1371/journal.pcbi.1000790)
Supplement: Figure S3 — Number of signatures output by TIE* as sample size grows. (0.01 MB PDF) [file pcbi.1000790.s003.pdf]

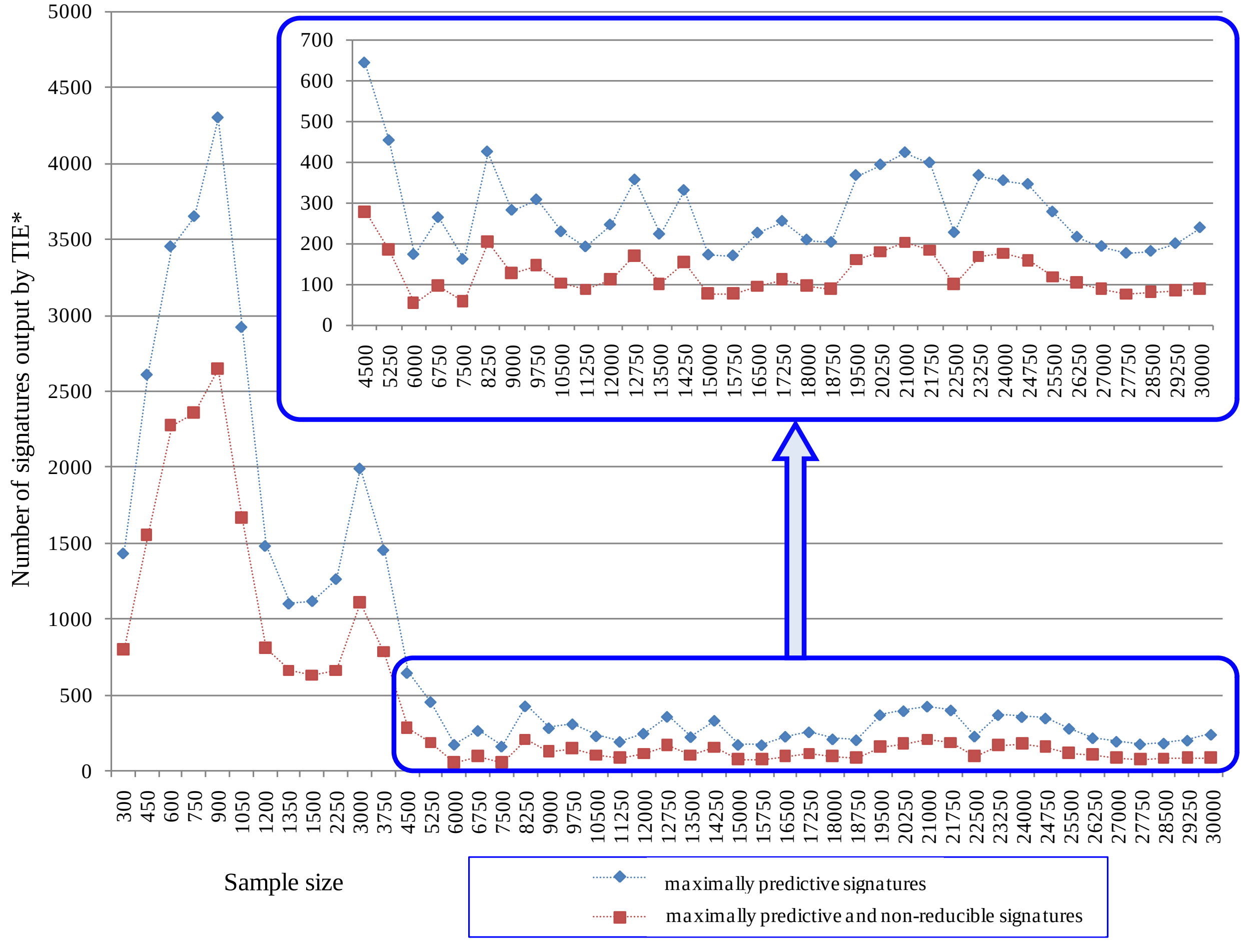

Figure S3: Number of signatures output by TIE* as sample size grows. The inner figure is a magnified region of the main figure.
