# Supplementary material for: Analysis and Computational Dissection of Molecular Signature Multiplicity
Source: PLoS Comput Biol. 2010 May 20;6(5):e1000790. doi: 10.1371/journal.pcbi.1000790 (PMC2873900; doi:10.1371/journal.pcbi.1000790)
Supplement: Table S1 — Gene expression microarray datasets used in this work. (0.03 MB PDF) [file pcbi.1000790.s004.pdf]

Task	*Discovery dataset*					*Validation dataset*					**Number of common genes**
	**Reference**	**Sample size**	**Samples per class**	**Number of genes**	**Microarray platform**	**Reference**	**Sample size**	**Samples per class**	**Number of genes**	**Microarray platform**	
***Lung Cancer Diagnosis*:** lung tumors vs. normals (non-tumor lung samples)	[1]	203	lung tumors (**186**)<br>normals (**17**)	12600	Affymetrix U95A	[2]	96	lung tumors (**86**)<br>normals (**10**)	7129	Affymetrix HuGeneFL	7094
***Lung Cancer Subtype Classification*:** adenocarcinoma vs. squamous cell carcinoma lung tumors	[1]	160	adenocarcinoma (**139**)<br>squamous (**21**)	12600	Affymetrix U95A	[3]	28	adenocarcinoma (**14**)<br>squamous (**14**)	12533	Affymetrix U95A	12533
***Breast Cancer Subtype Classification*:** estrogen receptor positive (ER+) vs. ER- breast tumors; untreated patients	[4]	286	ER+ (**209**)<br>ER- (**77**)	22283	Affymetrix U133A	[5]	119	ER+ (**85**)<br>ER- (**34**)	22283	Affymetrix U133A	22283
***Breast Cancer 5 Yr. Prognosis*:** ER+ patients who developed distant metastases within 5 years (poor prognosis) vs. ones who did not (good prognosis)	[4]	204	poor prognosis (**66**)<br>good prognosis (**138**)	22283	Affymetrix U133A	[5]	72	poor prognosis (**13**)<br>good prognosis (**59**)	22283	Affymetrix U133A	22283
***Glioma Subtype Classification*:** grade III vs. grade IV glioma tumors	[6]	100	grade III (**24**)<br>grade IV (**76**)	22283	Affymetrix U133A	[7]	85	grade III (**26**)<br>grade IV (**59**)	22283	Affymetrix U133A	22283
***Leukemia 5 Yr. Prognosis*:** patients with disease-free survival < 5 years (ones who had relapse or competing events within 5 years) vs. > 5 years	[8]	164	survival < 5 yr. (**29**)<br>survival > 5 yr. (**135**)	12625	Affymetrix U95A	[9]	79	survival < 5 yr. (**18**)<br>survival > 5 yr. (**61**)	22283	Affymetrix U133A	10507

Table S1: Gene expression microarray datasets used in this work.

References

1. Bhattacharjee A, Richards WG, Staunton J, Li C, Monti S, Vasa P, Ladd C, Beheshti J, Bueno R, Gillette M, Loda M, Weber G, Mark EJ, Lander ES, Wong W, Johnson BE, Golub TR, Sugarbaker DJ, Meyerson M (2001) Classification of human lung carcinomas by mRNA expression profiling reveals distinct adenocarcinoma subclasses. Proc Natl Acad Sci U S A 98: 13790-13795.

2. Beer DG, Kardia SL, Huang CC, Giordano TJ, Levin AM, Misek DE, Lin L, Chen G, Gharib TG, Thomas DG, Lizyness ML, Kuick R, Hayasaka S, Taylor JM, Iannettoni MD, Orringer MB, Hanash S (2002) Gene-expression profiles predict survival of patients with lung adenocarcinoma. Nat Med 8: 816-824.

3. Su AI, Welsh JB, Sapinoso LM, Kern SG, Dimitrov P, Lapp H, Schultz PG, Powell SM, Moskaluk CA, Frierson HF, Jr., Hampton GM (2001) Molecular classification of human carcinomas by use of gene expression signatures. Cancer Res 61: 7388-7393.

4. Wang Y, Klijn JG, Zhang Y, Sieuwerts AM, Look MP, Yang F, Talantov D, Timmermans M, Meijer-van Gelder ME, Yu J, Jatkoe T, Berns EM, Atkins D, Foekens JA (2005) Gene-expression profiles to predict distant metastasis of lymph-node-negative primary breast cancer. Lancet 365: 671-679.

5. Sotiriou C, Wirapati P, Loi S, Harris A, Fox S, Smeds J, Nordgren H, Farmer P, Praz V, Haibe-Kains B, Desmedt C, Larsimont D, Cardoso F, Peterse H, Nuyten D, Buyse M, van d, V, Bergh J, Piccart M, Delorenzi M (2006) Gene expression profiling in breast cancer: understanding the molecular basis of histologic grade to improve prognosis. J Natl Cancer Inst 98: 262-272.

6. Phillips HS, Kharbanda S, Chen R, Forrest WF, Soriano RH, Wu TD, Misra A, Nigro JM, Colman H, Soroceanu L, Williams PM, Modrusan Z, Feuerstein BG, Aldape K (2006) Molecular subclasses of high-grade glioma predict prognosis, delineate a pattern of disease progression, and resemble stages in neurogenesis. Cancer Cell 9: 157-173.

7. Freije WA, Castro-Vargas FE, Fang Z, Horvath S, Cloughesy T, Liau LM, Mischel PS, Nelson SF (2004) Gene expression profiling of gliomas strongly predicts survival. Cancer Res 64: 6503-6510.

8. Yeoh EJ, Ross ME, Shurtleff SA, Williams WK, Patel D, Mahfouz R, Behm FG, Raimondi SC, Relling MV, Patel A, Cheng C, Campana D, Wilkins D, Zhou X, Li J, Liu H, Pui CH, Evans WE, Naeve C, Wong L, Downing JR (2002) Classification, subtype discovery, and prediction of outcome in pediatric acute lymphoblastic leukemia by gene expression profiling. Cancer Cell 1: 133-143.

9. Ross ME, Zhou X, Song G, Shurtleff SA, Girtman K, Williams WK, Liu HC, Mahfouz R, Raimondi SC, Lenny N, Patel A, Downing JR (2003) Classification of pediatric acute lymphoblastic leukemia by gene expression profiling. Blood 102: 2951-2959.
