# Supplementary material for: Analysis and Computational Dissection of Molecular Signature Multiplicity
Source: PLoS Comput Biol. 2010 May 20;6(5):e1000790. doi: 10.1371/journal.pcbi.1000790 (PMC2873900; doi:10.1371/journal.pcbi.1000790)
Supplement: Table S2 — Results of experiments with artificial dataset with 30 variables. (0.02 MB PDF) [file pcbi.1000790.s005.pdf]

Method	Total number of output signatures	Number of variables in an average output signature	Number of true signatures		Average number of redundant variables in identified true signatures	Average classification performance in validation data	CPU time in minutes
			identified exactly	identified with redundant variables			
TIE*	72	5.00	72	72	0.00	0.951	0.39
Iterative Removal	3	4.67	0	1	1.00	0.946	0.01
KIAMB1	5000	2.83	0	0	N/A	0.776	11.55
KIAMB2	5000	2.82	0	0	N/A	0.772	11.69
KIAMB3	5000	2.81	0	0	N/A	0.774	11.62
Resampling+Univariate1	5000	17.87	0	72	12.00	0.949	84.56
Resampling+Univariate2	5000	7.54	0	25	12.12	0.924	85.50
Resampling+RFE1	5000	14.25	0	72	5.01	0.954	78.71
Resampling+RFE2	5000	5.80	1	44	4.25	0.939	79.26

Table S2: Results of experiments with artificial dataset with 30 variables. 72 true maximally predictive and non-redundant signatures exist in this dataset. The optimal Bayes classification performance is 0.9663 (weighted accuracy).
