# Supplementary material for: Analysis and Computational Dissection of Molecular Signature Multiplicity
Source: PLoS Comput Biol. 2010 May 20;6(5):e1000790. doi: 10.1371/journal.pcbi.1000790 (PMC2873900; doi:10.1371/journal.pcbi.1000790)
Supplement: Table S3 — Results of experiments with artificial dataset with 1,000 variables. (0.02 MB PDF) [file pcbi.1000790.s006.pdf]

Method	Total number of output signatures	Number of variables in an average output signature	Number of true signatures		Average number of redundant variables in identified true signatures	Average classification performance in validation data	CPU time in minutes
			identified exactly	identified with redundant variables			
TIE*	72	5.00	72	72	0.00	0.957	0.46
Iterative Removal	3	5.67	0	1	2.00	0.959	0.04
KIAMB1	5000	2.82	0	0	N/A	0.798	285.42
KIAMB2	5000	2.81	0	0	N/A	0.796	285.45
KIAMB3	5000	2.80	0	0	N/A	0.796	285.48
Resampling+Univariate1	5000	11.10	0	72	12.29	0.942	5999.64
Resampling+Univariate2	5000	5.58	0	0	N/A	0.934	6000.41
Resampling+RFE1	5000	8.70	0	72	6.38	0.952	6235.28
Resampling+RFE2	5000	4.24	0	29	5.76	0.947	6235.93

Table S3: Results of experiments with artificial dataset with 1,000 variables. 72 true maximally predictive and non-redundant signatures exist in this dataset.
