# Supplementary material for: Analysis and Computational Dissection of Molecular Signature Multiplicity
Source: PLoS Comput Biol. 2010 May 20;6(5):e1000790. doi: 10.1371/journal.pcbi.1000790 (PMC2873900; doi:10.1371/journal.pcbi.1000790)
Supplement: Table S4 — Results for the number of output signatures (total/unique/unique and non-reducible), number of genes in a signature, and classification performance in discovery and validation microarray datasets. (0.06 MB PDF) [file pcbi.1000790.s007.pdf]

Lung Cancer Diagnosis

Method to induce multiple signatures	Number of signatures	Number of genes in a signature		Classification performance (AUC)			
				In discovery dataset		In validation dataset	
		mean	95% interval	mean	95% interval	mean	95% interval
TIE*	348/348/187	6	[4 - 8]	0.999	[0.994 - 1.000]	0.998	[0.988 - 1.000]
Resampling+SVM-RFE1	5000/2966/48	9	[1 - 43]	0.987	[0.919 - 1.000]	0.989	[0.949 - 1.000]
Resampling+SVM-RFE2	5000/341/61	1	[1 - 2]	0.967	[0.861 - 1.000]	0.962	[0.633 - 1.000]
Resampling+Univariate1	5000/2199/19	19	[1 - 62]	0.99	[0.919 - 1.000]	0.992	[0.949 - 1.000]
Resampling+Univariate2	5000/294/58	1	[1 - 2]	0.969	[0.861 - 1.000]	0.973	[0.887 - 1.000]
KIAMB1	985/985/985	41	[39 - 42]	0.999	[0.990 - 1.000]	0.995	[0.984 - 1.000]
KIAMB2	1489/1320/1246	48	[12 - 68]	0.999	[0.990 - 1.000]	0.995	[0.978 - 1.000]
KIAMB3	5000/271/157	9	[6 - 15]	0.996	[0.981 - 1.000]	0.997	[0.992 - 1.000]
Iterative Removal	51/51/51	7	[5 - 10]	0.987	[0.919 - 1.000]	0.977	[0.880 - 1.000]

Lung Cancer Subtype Classification

Method to induce multiple signatures	Number of signatures	Number of genes in a signature		Classification performance (AUC)			
				In discovery dataset		In validation dataset	
		mean	95% interval	mean	95% interval	mean	95% interval
TIE*	668/668/413	7	[5 - 8]	0.987	[0.973 - 1.000]	0.973	[0.929 - 1.000]
Resampling+SVM-RFE1	5000/4267/20	392	[1 - 5037]	0.98	[0.909 - 1.000]	0.978	[0.888 - 1.000]
Resampling+SVM-RFE2	5000/1206/107	2	[1 - 5]	0.925	[0.650 - 0.985]	0.914	[0.668 - 0.995]
Resampling+Univariate1	5000/4590/55	528	[1 - 8703]	0.98	[0.903 - 1.000]	0.98	[0.883 - 1.000]
Resampling+Univariate2	5000/917/81	3	[1 - 6]	0.922	[0.839 - 0.988]	0.916	[0.770 - 0.985]
KIAMB1	994/968/965	26	[24 - 26]	0.986	[0.967 - 1.000]	0.982	[0.923 - 1.000]
KIAMB2	1006/1005/1005	48	[47 - 50]	0.99	[0.973 - 1.000]	0.982	[0.923 - 1.000]
KIAMB3	3520/1364/1209	16	[8 - 31]	0.98	[0.948 - 0.997]	0.982	[0.923 - 1.000]
Iterative Removal	29/29/29	8	[5 - 12]	0.978	[0.867 - 1.000]	0.972	[0.882 - 1.000]

Breast Cancer Subtype Classification

Method to induce multiple signatures	Number of signatures	Number of genes in a signature		Classification performance (AUC)			
				In discovery dataset		In validation dataset	
		mean	95% interval	mean	95% interval	mean	95% interval
TIE*	2776/2776/1602	17	[14 - 21]	0.847	[0.824 - 0.873]	0.887	[0.852 - 0.916]
Resampling+SVM-RFE1	5000/4601/22	1627	[1 - 10746]	0.845	[0.821 - 0.888]	0.812	[0.604 - 0.893]
Resampling+SVM-RFE2	5000/2033/65	18	[1 - 135]	0.858	[0.736 - 0.930]	0.761	[0.554 - 0.874]
Resampling+Univariate1	5000/4122/15	3560	[1 - 22283]	0.857	[0.826 - 0.920]	0.823	[0.771 - 0.877]
Resampling+Univariate2	5000/794/22	7	[1 - 18]	0.873	[0.754 - 0.930]	0.814	[0.725 - 0.874]
KIAMB1	983/970/960	31	[30 - 32]	0.85	[0.804 - 0.883]	0.68	[0.427 - 0.846]
KIAMB2	994/964/962	28	[27 - 29]	0.85	[0.802 - 0.884]	0.685	[0.418 - 0.850]
KIAMB3	943/570/493	14	[12 - 15]	0.856	[0.786 - 0.884]	0.694	[0.432 - 0.851]
Iterative Removal	34/34/34	19	[14 - 23]	0.833	[0.793 - 0.866]	0.834	[0.720 - 0.899]

Table S4 (continued on the next page): Results for the number of output signatures (total/unique/unique and non-reducible), number of genes in a signature, and classification performance in discovery and validation microarray datasets. The length of highlighting corresponds to magnitude of the value (number of genes in a signature or classification performance) relative to other multiple signature extraction methods. The 95% intervals correspond to the observed [2.5 - 97.5] percentile interval over multiple signatures discovered by the method. Uniqueness and non-reducibility of each signature is assessed relative to the output of the corresponding signature extraction method.

Breast Cancer 5 Yr. Prognosis

Method to induce multiple signatures	Number of signatures	Number of genes in a signature		Classification performance (AUC)			
				In discovery dataset		In validation dataset	
		mean	95% interval	mean	95% interval	mean	95% interval
TIE*	5342/5342/3321	84	[81 - 89]	0.671	[0.658 - 0.686]	0.697	[0.674 - 0.720]
Resampling+SVM-RFE1	5000/4755/42	4687	[2 - 22283]	0.684	[0.541 - 0.746]	0.64	[0.487 - 0.752]
Resampling+SVM-RFE2	5000/3407/350	56	[1 - 404]	0.586	[0.413 - 0.719]	0.598	[0.398 - 0.822]
Resampling+Univariate1	5000/4002/29	5791	[1 - 22283]	0.685	[0.573 - 0.741]	0.645	[0.468 - 0.801]
Resampling+Univariate2	5000/2573/139	44	[1 - 162]	0.62	[0.467 - 0.712]	0.628	[0.411 - 0.807]
KIAMB1	986/552/550	14	[14 - 14]	0.596	[0.507 - 0.693]	0.562	[0.399 - 0.716]
KIAMB2	988/969/955	28	[27 - 29]	0.595	[0.482 - 0.708]	0.562	[0.390 - 0.713]
KIAMB3	1182/916/889	23	[12 - 28]	0.596	[0.483 - 0.704]	0.567	[0.394 - 0.724]
Iterative Removal	31/31/31	28	[12 - 82]	0.69	[0.589 - 0.794]	0.606	[0.434 - 0.735]

Glioma Subtype Classification

Method to induce multiple signatures	Number of signatures	Number of genes in a signature		Classification performance (AUC)			
				In discovery dataset		In validation dataset	
		mean	95% interval	mean	95% interval	mean	95% interval
TIE*	5753/5753/4588	46	[45 - 53]	0.871	[0.860 - 0.885]	0.844	[0.830 - 0.860]
Resampling+SVM-RFE1	5000/4255/43	301	[2 - 3599]	0.808	[0.630 - 0.915]	0.74	[0.528 - 0.880]
Resampling+SVM-RFE2	5000/2055/126	3	[1 - 13]	0.694	[0.545 - 0.890]	0.637	[0.463 - 0.830]
Resampling+Univariate1	5000/4751/63	925	[2 - 17022]	0.84	[0.690 - 0.905]	0.818	[0.554 - 0.919]
Resampling+Univariate2	5000/1926/117	3	[1 - 15]	0.74	[0.495 - 0.900]	0.65	[0.450 - 0.860]
KIAMB1	973/658/654	15	[15 - 15]	0.765	[0.675 - 0.865]	0.71	[0.558 - 0.811]
KIAMB2	974/964/964	30	[29 - 30]	0.781	[0.685 - 0.880]	0.732	[0.610 - 0.832]
KIAMB3	1408/786/746	21	[6 - 30]	0.77	[0.685 - 0.865]	0.728	[0.588 - 0.821]
Iterative Removal	58/58/58	24	[15 - 44]	0.847	[0.744 - 0.921]	0.842	[0.743 - 0.914]

Leukemia 5 Yr. Prognosis

Method to induce multiple signatures	Number of signatures	Number of genes in a signature		Classification performance (AUC)			
				In discovery dataset		In validation dataset	
		mean	95% interval	mean	95% interval	mean	95% interval
TIE*	1804/1804/1561	22	[20 - 28]	0.714	[0.647 - 0.805]	0.711	[0.631 - 0.784]
Resampling+SVM-RFE1	5000/4643/158	1984	[1 - 8756]	0.631	[0.422 - 0.741]	0.612	[0.440 - 0.725]
Resampling+SVM-RFE2	5000/2537/570	15	[1 - 92]	0.543	[0.341 - 0.749]	0.55	[0.356 - 0.725]
Resampling+Univariate1	5000/3897/116	4024	[1 - 10507]	0.649	[0.431 - 0.756]	0.606	[0.419 - 0.717]
Resampling+Univariate2	5000/2516/465	48	[1 - 329]	0.539	[0.235 - 0.756]	0.529	[0.342 - 0.725]
KIAMB1	988/984/984	31	[29 - 31]	0.515	[0.351 - 0.681]	0.603	[0.445 - 0.735]
KIAMB2	1213/1131/1127	46	[13 - 56]	0.517	[0.341 - 0.687]	0.602	[0.460 - 0.732]
KIAMB3	4485/30/30	7	[6 - 10]	0.438	[0.348 - 0.632]	0.563	[0.530 - 0.760]
Iterative Removal	2/2/2	21	[19 - 23]	0.673	[0.630 - 0.716]	0.652	[0.550 - 0.753]

Table S4 (continued from the previous page)
