# Supplementary material for: Analysis and Computational Dissection of Molecular Signature Multiplicity
Source: PLoS Comput Biol. 2010 May 20;6(5):e1000790. doi: 10.1371/journal.pcbi.1000790 (PMC2873900; doi:10.1371/journal.pcbi.1000790)
Supplement: Table S5 — Number of common genes in 50%, 60%, …, 100% of signatures discovered by TIE* algorithm for each dataset. (0.04 MB PDF) [file pcbi.1000790.s008.pdf]

Task	Number of genes common in X% of discovered signatures					
	50%	60%	70%	80%	90%	100%
Lung Cancer Diagnosis	4	0	0	0	0	0
Lung Cancer Subtype Classification	5	0	0	0	0	0
Breast Cancer Subtype Classification	15	11	4	1	0	0
Breast Cancer 5 Yr. Prognosis	85	85	84	84	84	1
Glioma Subtype Classification	48	48	48	47	41	0
Leukemia 5 Yr. Prognosis	23	23	23	20	1	0

Table S5: Number of common genes in 50%, 60%, …, 100% of signatures discovered by TIE* algorithm for each dataset.
