# Supplementary material for: Analysis and Computational Dissection of Molecular Signature Multiplicity
Source: PLoS Comput Biol. 2010 May 20;6(5):e1000790. doi: 10.1371/journal.pcbi.1000790 (PMC2873900; doi:10.1371/journal.pcbi.1000790)
Supplement: Text S1 — Maximally predictive and non-redundant molecular signatures are precisely the Markov boundaries and vice-versa. (0.07 MB PDF) [file pcbi.1000790.s009.pdf]

Maximally predictive and non-redundant molecular signatures are precisely the Markov boundaries and vice-versa

In the present paper capital letters in italics denote variables (e.g., *A*, *B*, *C*) and bold letters denote variable sets (e.g., **X**, **Y**, **Z**). We also adopt the following standard notation of statistical independence relations: $T \perp \mathbf{A}$ means that *T* is independent of the variable set **A**. Similarly, if *T* is independent of the variable set **A** conditioned the variable set **B**, we denote this as $T \perp \mathbf{A} \mid \mathbf{B}$. If we use the sign "$\not\perp$" instead of "$\perp$", this means dependence instead of independence.

Now we introduce several key definitions:

- Molecular signature: A molecular signature is a mathematical/computational model (e.g., classifier or regression model) that predicts a phenotypic response variable of interest *T* (e.g., diagnosis or response to treatment in human patients) given values of molecular variables (e.g., gene expression values).
- Maximally predictive molecular signature: A maximally predictive molecular signature is a molecular signature that maximizes predictivity of the phenotypic response variable *T* relative to all other signatures that can be constructed from the given dataset.
- Maximally predictive and non-redundant molecular signature: A maximally predictive and non-redundant molecular signature based on variables **X** is a maximally predictive signature such that any signature based on a proper subset of variables in **X** is not maximally predictive.
- Markov blanket: A Markov blanket **M** of the response variable $T \in \mathbf{V}$ in the joint probability distribution $\mathbb{P}$ over variables **V** is a set of variables conditioned on which all other variables are independent of *T*, i.e. for every $X \in (\mathbf{V} \setminus \mathbf{M} \setminus \{T\})$, $T \perp X \mid \mathbf{M}$.
- Market boundary: If **M** is a Markov blanket of *T* and no proper subset of **M** satisfies the definition of Markov blanket of *T*, then **M** is called a Markov boundary of *T*.

Theorem: If $\mathbb{W}$ is a performance metric that is maximized only when P(*T* | **V** \ {*T*}) is estimated accurately and $\mathbb{L}$ is a learning algorithm that can approximate any probability distribution, then **M** is a Markov blanket of *T* if and only if the learner's model induced using variables **M** is a maximally predictive signature of *T*.

Proof: First we prove that the learner's model induced using any Markov blanket of *T* is a maximally predictive signature of *T*. If **M** is Markov blanket of *T*, then by definition it leads to a maximally predictive signature of *T* because P(*T* | **M**) = P(*T* | **V** \ {*T*}) and this distribution can be perfectly approximated by $\mathbb{L}$, which implies that $\mathbb{W}$ will be maximized. Now we prove that any maximally predictive signature of *T* is the learner's model induced using a Markov blanket of *T*. Assume that $\mathbf{X} \subseteq \mathbf{V} \setminus \{T\}$ is a set of variables used in the maximally predictive signature of *T* but it is not a Markov blanket of *T*. This implies that, P(*T* | **X**) ≠ P(*T* | **V** \ {*T*}). By definition of the Markov blanket, **V** \ {*T*} is always a Markov blanket of *T*. By first part of the theorem, **V** \ {*T*} leads to a maximally predictive signature of *T* similarly to **X**. Therefore, the following should hold: P(*T* | **X**) = P(*T* | **V** \ {*T*}). This contradicts the assumption that **X** is not a Markov blanket of *T*. Therefore, **X** is a Markov blanket of *T*. (Q.E.D.)

Since the notion of non-redundancy is defined in the same way for maximally predictive signatures and for Markov blankets, under the assumptions of the above theorem it follows that **M** is a Markov boundary of *T* if and only if the learner's model induced using variables **M** is a maximally predictive and non-redundant signature of *T*.
