# Supplementary material for: Analysis and Computational Dissection of Molecular Signature Multiplicity
Source: PLoS Comput Biol. 2010 May 20;6(5):e1000790. doi: 10.1371/journal.pcbi.1000790 (PMC2873900; doi:10.1371/journal.pcbi.1000790)
Supplement: Text S2 — An example of signature multiplicity due to small samples. (0.08 MB PDF) [file pcbi.1000790.s010.pdf]

An example of signature multiplicity due to small samples

Consider a simplified pathway structure and parameterization shown in the figure below. It involves 4 genes (*A*, *B*, *C*, *D*) and a phenotypic response variable *T*. This network encodes a faithful distribution[1] and thus only one Markov boundary exists in large samples [1–4], which is {*C*, *D*}. Now consider that we have access to three small samples from this distribution such that: in sample #1 one cannot reliably establish that $T \perp A \mid \{C,D\}$, in sample #2 one cannot reliably establish that $T \perp B \mid \{C,D\}$, and in sample #3 one cannot reliably establish either $T \perp A \mid \{C,D\}$ or $T \perp B \mid \{C,D\}$. Three Markov boundaries can be identified in the above samples, {*C*, *D*, *A*}, {*C*, *D*, *B*}, and {*C*, *D*, *A*, *B*}, respectively, assuming that neither *A* nor *B* significantly decreases the predictivity of *T* in given samples.

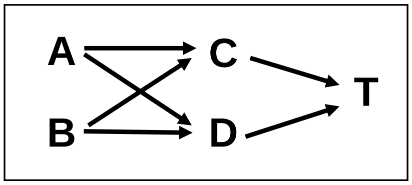

P(*T* | *C*, *D*)	(*C* = 0, *D* = 0)	(*C* = 0, *D* = 1)	(*C* = 1, *D* = 0)	(*C* = 1, *D* = 1)
T = 0	0.2	0.5	0.7	0.4
T = 1	0.8	0.5	0.3	0.6

P(*C* | *A*, *B*)	(*A* = 0, *B* = 0)	(*A* = 0, *B* = 1)	(*A* = 1, *B* = 0)	(*A* = 1, *B* = 1)
C = 0	0.3	0.7	0.9	0.4
C = 1	0.7	0.3	0.1	0.6

P(*D* | *A*, *B*)	(*A* = 0, *B* = 0)	(*A* = 0, *B* = 1)	(*A* = 1, *B* = 0)	(*A* = 1, *B* = 1)
D = 0	0.6	0.7	0.8	0.4
D = 1	0.4	0.3	0.2	0.6

P(*A*)	
A = 0	0.6
A = 1	0.4

P(*B*)	
B = 0	0.4
B = 1	0.6

Figure: Example pathway structure with 4 gene variables (*A*, *B*, *C*, *D*) and phenotypic response variable *T*. The structure is represented by a Bayesian network. The network parameterization is defined below the graph. All variables take values {0,1}.

[1] Let G be a graph and P a probability distribution. We will call a distribution P faithful to G if and only if every conditional independence relation true in P is entailed by the Markov Condition applied to G. In other words, the graph G using the Markov Condition provides an accurate map of all conditional and marginal dependencies and independencies in P. Recall that a probability distribution P satisfies the Markov Condition with respect to a graph G over variables **V** if for every variable *W* in **V**, *W* is independent of all non-descendants of *W* given the parents of *W* [1].

References

1. Spirtes, P., Glymour, C. N., and Scheines, R. (2000) Causation, prediction, and search. Cambridge, Mass: MIT Press.

2. Pearl, J. (1988) Probabilistic reasoning in intelligent systems: networks of plausible inference. San Mateo, California: Morgan Kaufmann Publishers.

3. Pearl, J. (2000) Causality: models, reasoning, and inference. Cambridge, U.K: Cambridge University Press.

4. Tsamardinos I, Aliferis CF (2003) Towards principled feature selection: relevancy, filters and wrappers. Proceedings of the Ninth International Workshop on Artificial Intelligence and Statistics (AI & Stats) .
