# Supplementary material for: Analysis and Computational Dissection of Molecular Signature Multiplicity
Source: PLoS Comput Biol. 2010 May 20;6(5):e1000790. doi: 10.1371/journal.pcbi.1000790 (PMC2873900; doi:10.1371/journal.pcbi.1000790)
Supplement: Text S3 — Details about the TIE* algorithm. (0.15 MB PDF) [file pcbi.1000790.s011.pdf]

Details about the TIE* algorithm

Trace of the algorithm

Consider running TIE* algorithm in the example pathway structure depicted in the figure below. The phenotypic response variable T is caused by 4 genes: C, D, E, and F. The distribution is such that genes A and C contain exactly the same information about T; likewise two genes $\{D, E\}$ jointly and a single gene B contain exactly the same information about T. In step 1 of TIE* (Figure 2 in the main manuscript), the algorithm identifies a Markov boundary $\mathbf{M} = \{A, B, F\}$ and outputs it. In step 3, the algorithm generates a subset $\mathbf{G} = \{F\}$ of the previously identified Markov boundaries. Then in step 4 the base algorithm is run on all genes but F. This yields a candidate Markov boundary $\mathbf{M}_{new} = \{A, B\}$ that has suboptimal predictivity of the phenotype as determined in step 5, and thus it is not a Markov boundary. Then in step 3 the algorithm generates another subset $\mathbf{G} = \{A\}$. The base algorithm in step 4 yields a new candidate Markov boundary $\mathbf{M}_{new} = \{C, B, F\}$ that as determined in step 5 has the same predictivity of the phenotype T as $\mathbf{M}$. Therefore $\mathbf{M}_{new}$ is indeed a Markov boundary and it is output in step 5. Similarly, when the base algorithm is run on data for all genes but $\mathbf{G} = \{B\}$ and $\mathbf{G} = \{A, B\}$, two more Markov boundaries $\{A, D, E, F\}$ and $\{C, D, E, F\}$, are found and output. The algorithm terminates shortly. In total, four Markov boundaries are output by the algorithm: $\{A, B, F\}$, $\{C, B, F\}$, $\{A, D, E, F\}$ and $\{C, D, E, F\}$. These are exactly all the Markov boundaries that exist in this distribution.

Proof of correctness

<u>Theorem:</u> TIE* outputs all and only the Markov boundaries of the response variable T if the following five conditions hold:

(1) The base algorithm correctly identifies a Markov boundary of T both in the original data and in every modified version of the data that results after removing a subset of variables in step 3 of the algorithm (so-called "embedded" distribution).
(2) The learning algorithm to fit a predictive model of the response variable T given a set of predictor variables can accurately model the conditional probability distribution of T given the set of predictor variables.
(3) The performance metric to assess predictivity of the signatures is maximized only when the conditional probability of T given all other variables is accurately estimated.
(4) The procedure to estimate predictive error of the signatures is unbiased.
(5) The procedure to compare estimates of predictive error of the signatures has negligible error.

Proof: First we prove that condition (ii) in step 3 of TIE* (Figure 2 in the main manuscript) does not affect output of the algorithm. This result is important because it simplifies further proof of correctness and also provides a justification for the computational savings in TIE* incurred by using this condition. We prove this result by contradiction. Consider that the algorithm has previously discovered a Markov boundary $\mathbf{M}_i$ and a subset $\mathbf{G}' \subset \mathbf{M}_i$ is generated in step 3 such that the resulting candidate Markov boundary $\mathbf{M}_{new}$ in step 4 is not a Markov boundary in the original distribution. Since removal of $\mathbf{G}'$ leads to a non-Markov boundary in the original distribution, the algorithm does not generate supersets of $\mathbf{G}'$ in step 3 according to condition (ii).

Assume that there is a Markov boundary $\mathbf{M}_j$ that is not output by the TIE* algorithm because a subset of variables $\mathbf{G}'' = \mathbf{M}_i \supset \mathbf{G}'$ was not generated in step 3. This implies that predictivity of $\mathbf{M}_j$ of the phenotype is larger than one of $\mathbf{M}_{new}$. Therefore, $\mathbf{M}_{new}$ is not a Markov boundary in the embedded distribution after removing a subset of variables $\mathbf{G}'$. This contradicts assumption (1) of the theorem. *Therefore, condition (ii) in step 3 of TIE* does not affect output of the algorithm and we can proceed with further theoretical analysis as if this condition does not exist.*

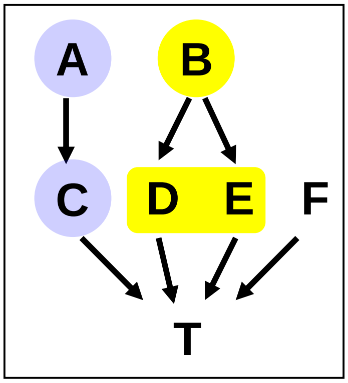

P(A)	
A = 0	0.6
A = 1	0.4

P(B)	
B = 0	0.3
B = 1	0.2
B = 2	0.3
B = 3	0.2

P(C | A)	A = 0	A = 1
C = 0	0.0	1.0
C = 1	1.0	0.0

P(F)	
F = 0	0.3
F = 1	0.7

P(D | B)	B = 0	B = 1	B = 2	B = 3
D = 0	1.0	1.0	0.0	0.0
D = 1	0.0	0.0	1.0	1.0

P(E | B)	B = 0	B = 1	B = 2	B = 3
E = 0	1.0	0.0	1.0	0.0
E = 1	0.0	1.0	0.0	1.0

P(T | C, D, E, F)	(C=0, D=0, E=0, F=0)	(C=0, D=0, E=0, F=1)	(C=0, D=0, E=1, F=0)	…	(C=1, D=1, E=1, F=1)
T = 0	0.9	0.1	0.9		0.1
T = 1	0.1	0.9	0.1		0.9

Figure: Example pathway structure with 6 gene variables (A, B, C, D, E, F) and phenotypic response variable T. The structure is represented by a Bayesian network. The network parameterization is defined below the graph. All variables take values {0,1} except for B that takes values {0,1,2,3}. Genes A and C contain exactly the same information about T and are highlighted with the same color. Likewise two genes {D, E} jointly and a single gene B contain exactly the same information about T and thus are also highlighted with the same color.

- Assume that TIE* returns **X** that is not a Markov boundary of *T*. Then **X** is not a Markov blanket of *T* because of condition (1) and the fact that non-redundancy property of **X** holds in every embedded distribution. **X** cannot coincide with the variable set **M** identified in step 1 because it is a Markov boundary of *T* in the original distribution according to (1). Thus, it has to be a set of variables $\mathbf{M}_{new}$ identified in step 4. However, for $\mathbf{M}_{new}$ to be output it should have the same predictivity of the phenotype as the Markov boundary **M**. Given assumptions (2) - (5), this can happen if and only if $\mathbf{M}_{new}$ is a Markov blanket. Thus, we have reached a contradiction and we conclude that *TIE* would never output variable sets that are not Markov boundaries*.
- Assume that there exists a Markov boundary **X** that is not output by TIE*. Because of assumptions (2) - (5), $\mathbf{M}_{new}$ = **X** was never identified in step 4 of the algorithm. This can happen if and only if $T \perp \mathbf{X}|\mathbf{M}_i$ where $\mathbf{M}_i$ is a Markov boundary that was previously discovered by TIE* (either in step 1 or 4). However, in some iteration of the TIE* algorithm, the set **G** = $\mathbf{M}_i$ (and similarly other sets that render **X** independent of *T*) will be generated in step 3 and $\mathbf{M}_{new}$ = **X** will be identified in step 4 after removing **G** from the data. Therefore, we have reached a contradiction and we conclude that *TIE* would never miss Markov boundaries*. (Q.E.D.)

The following lemma provides an instantiation of the TIE* algorithm that does not require learning of the predictive model of the phenotypic response variable *T*.

<u>Lemma:</u> Assume that a set of variables $\mathbf{M} \subseteq (\mathbf{V} \setminus \{T\})$ is a Markov boundary of *T* (**V** denotes all variables in the network). If there exists a set of variables $\mathbf{M}_{new} \subseteq (\mathbf{V} \setminus \{T\})$ such that $T \perp \mathbf{M} \mid \mathbf{M}_{new}$ and $\mathbf{M}_{new}$ is a Markov boundary of T in the embedded distribution, then $\mathbf{M}_{new}$ is also a Markov boundary of *T* in the original distribution.

Proof: The proof below makes references to several properties of probability distributions that are given in [1]. Consider that there exists a set of variables $\mathbf{M}_{new} \subseteq \mathbf{V} \setminus \{T\}$ such that $T \perp \mathbf{M} \mid \mathbf{M}_{new}$. Since **M** is a Markov boundary of *T* in the original distribution, it is also a Markov blanket of *T* in the original distribution. By definition of the Markov blanket, $T \perp (\mathbf{V} \setminus \mathbf{M} \setminus \{T\}) \mid \mathbf{M}$. By self-conditioning property, it follows that $T \perp (\mathbf{V} \setminus \{T\}) \mid \mathbf{M}$. Since $(\mathbf{V} \setminus \{T\}) = (\mathbf{V} \setminus \{T\}) \cup \mathbf{M}_{new}$ and according to the weak union property, $T \perp (\mathbf{V} \setminus \{T\} \setminus \mathbf{M}_{new}) \mid (\mathbf{M} \cup \mathbf{M}_{new})$. By self-conditioning property, it follows that $T \perp (\mathbf{V} \setminus \{T\}) \mid (\mathbf{M} \cup \mathbf{M}_{new})$. Since $T \perp \mathbf{M} \mid \mathbf{M}_{new}$ and $T \perp (\mathbf{V} \setminus \{T\}) \mid (\mathbf{M} \cup \mathbf{M}_{new})$, the contraction property implies that $T \perp ((\mathbf{V} \setminus \{T\}) \cup \mathbf{M}) \mid \mathbf{M}_{new}$. Since $(\mathbf{V} \setminus \{T\}) = (\mathbf{V} \setminus \{T\}) \cup \mathbf{M}$, it follows that $T \perp (\mathbf{V} \setminus \{T\}) \mid \mathbf{M}_{new}$. By decomposition property this implies that $\mathbf{M}_{new}$ is a Markov blanket of *T* in the original distribution. Since $\mathbf{M}_{new}$ is a Markov boundary of *T* in the embedded distribution and it is a Markov blanket of *T* in the original distribution, it is also a Markov boundary of *T* in the original distribution. (Q.E.D.)

The above lemma suggests a new step 5 in the TIE* algorithm (see Figure 2 in the main manuscript): If $T \perp \mathbf{M}|\mathbf{M}_{new}$, then $\mathbf{M}_{new}$ is indeed a Markov boundary, and it is output.

A more detailed theoretical analysis of the generative TIE* algorithm and the set of concrete sound instantiations is provided in [2].

Algorithm implementation details

In experiments with real and/or resimulated gene expression data, we used the following implementation of TIE*:

- To identify Markov boundaries, we used the base algorithm HITON-PC (Figure S2) with Fisher's *Z*-test [3,4]. The parameters α and *max-k* of the algorithm were optimized by holdout validation[1] to achieve maximum predictivity of the phenotypic response variable.
- To fit classification models of the phenotypic response variable using identified gene sets, we used SVM classifiers [5].
- To estimate predictivity of signatures, we used holdout validation method whereby 2/3 of the data was used to identify genes in the signatures and fit the classifier and 1/3 of the data was used to estimate classification performance using AUC metric [6,7].
- To compare predictivity of signatures, we used the nonparametric method of Delong et al. [8].
- Finally, to run TIE* efficiently we constrained the cardinality of the subset **G** in step 3 of the algorithm, thus trading off completeness for execution speed.

In experiments with simulated data where both the generative model and all the true Markov boundaries are known, we used a similar implementation of TIE* with the following two differences. *First*, instead of Fisher's Z-test, we used G^2 test that is suitable for the distribution at hand. *Second*, we did not estimate predictivity to verify that a new candidate Markov boundary $\mathbf{M}_{new}$ is a Markov boundary in the original distribution. To establish this, we directly applied the lemma described above. This allowed avoiding potential errors in predictivity estimation and increase effective sample size (since we did not have to split the data into training and testing sets), thus improving overall performance of the algorithm. However, we did not adopt this strategy for other experiments where the sample size was typically much smaller and the high-dimensional conditioning tests used in the above lemma were unreliable.

Classification methods

To build classification models of the phenotype from identified gene sets, we used the support vector machine (SVM) algorithm [5] that is known to be the "best of class" method for classification of gene expression microarray data [9–11]. The underlying idea of SVM classifiers is to calculate a maximal margin hyperplane separating two categories of subjects, e.g., cases and controls. Subjects are classified according to the side of the hyperplane they belong to. We used the SVM implementation in the *libSVM* software library [12]. For experiments with artificial data where the response variable is multicategorical, we used one-versus-rest SVM classifiers [13].

[1] The parameters are optimized over the following values: (α = 0.05, *max-k* = 1), (α = 0.05, *max-k* = 2), (α = 0.05, *max-k* = 3), (α = 0.01, *max-k* = 1), and (α = 0.01, *max-k* = 2).

Metrics for assessing predictivity

For experiments with real and resimulated gene expression data where the phenotypic response variable had two categories, we used area under ROC curve (AUC) metric [6]. For experiments with artificial simulated data where the response variable was multicategorical, we used weighted accuracy [14].

References

1. Pearl, J. (1988) Probabilistic reasoning in intelligent systems: networks of plausible inference. San Mateo, California: Morgan Kaufmann Publishers.

2. Statnikov A (2008) Algorithms for Discovery of Multiple Markov Boundaries: Application to the Molecular Signature Multiplicity Problem. Ph D Thesis, Department of Biomedical Informatics, Vanderbilt University .

3. Aliferis CF, Statnikov A, Tsamardinos I, Mani S, Koutsoukos XD (2010) Local Causal and Markov Blanket Induction for Causal Discovery and Feature Selection for Classification. Part II: Analysis and Extensions. Journal of Machine Learning Research 11: 235-284.

4. Aliferis CF, Statnikov A, Tsamardinos I, Mani S, Koutsoukos XD (2010) Local Causal and Markov Blanket Induction for Causal Discovery and Feature Selection for Classification. Part I: Algorithms and Empirical Evaluation. Journal of Machine Learning Research 11: 171-234.

5. Vapnik, V. N. (1998) Statistical learning theory. New York: Wiley.

6. Fawcett T (2003) ROC Graphs: Notes and Practical Considerations for Researchers. Technical Report, HPL-2003-4, HP Laboratories .

7. Weiss, S. M. and Kulikowski, C. A. (1991) Computer systems that learn: classification and prediction methods from statistics, neural nets, machine learning, and expert systems. San Mateo, Calif: M. Kaufmann Publishers.

8. DeLong ER, DeLong DM, Clarke-Pearson DL (1988) Comparing the areas under two or more correlated receiver operating characteristic curves: a nonparametric approach. Biometrics 44: 837-845.

9. Statnikov A, Aliferis CF, Tsamardinos I, Hardin D, Levy S (2005) A comprehensive evaluation of multicategory classification methods for microarray gene expression cancer diagnosis. Bioinformatics 21: 631-643.

10. Furey TS, Cristianini N, Duffy N, Bednarski DW, Schummer M, Haussler D (2000) Support vector machine classification and validation of cancer tissue samples using microarray expression data. Bioinformatics 16: 906-914.

11. Statnikov A, Wang L, Aliferis CF (2008) A comprehensive comparison of random forests and support vector machines for microarray-based cancer classification. BMC Bioinformatics 9: 319.

12. Fan RE, Chen PH, Lin CJ (2005) Working set selection using second order information for training support vector machines. Journal of Machine Learning Research 6: 1918.

13. Schölkopf, B., Burges, C. J. C., and Smola, A. J. (1999) Advances in kernel methods: support vector learning. Cambridge, Mass: MIT Press.

14. Guyon, I, Gunn, S, Nikravesh, M., and Zadeh, L. A. (2006) Feature extraction: foundations and applications. Berlin: Springer-Verlag.
