# Supplementary material for: Analysis and Computational Dissection of Molecular Signature Multiplicity
Source: PLoS Comput Biol. 2010 May 20;6(5):e1000790. doi: 10.1371/journal.pcbi.1000790 (PMC2873900; doi:10.1371/journal.pcbi.1000790)
Supplement: Text S4 — Previous algorithms for multiple signature identification used in experiments. (0.08 MB PDF) [file pcbi.1000790.s012.pdf]

Previous algorithms for multiple signature identification used in experiments

Eight previously described methods to extract multiple signatures and compare to TIE* were used in experiments. These algorithms were executed on Intel Xeon 2.4 GHz CPUs for up to one week of single-CPU time or to produce up to 5,000 signatures (per method and dataset), whatever termination criterion was met first.

Four methods were resampling-based techniques that apply a signature extraction algorithm to bootstrap samples of the original dataset. The following signature extraction algorithms were used: (i) SVM-based recursive feature elimination (SVM-RFE) [1]; (ii) SVM-RFE with additional application of a formal statistical comparison test[1] to identify the most parsimonious signature with predictivity statistically indistinguishable from the observed best one; (iii) backward wrapping based on univariate ranking of variables by Kruskal-Wallis non-parametric ANOVA [4,5]; and (iv) backward wrapping based on Kruskal-Wallis ANOVA with additional statistical comparison step, as in (ii). The above four methods are denoted as Resampling-SVM-RFE1, Resampling-SVM-RFE2, Resampling-Univariate1, Resampling-Univariate2, respectively.

Three other methods were representatives of stochastic variable selection algorithms. Specifically, three instantiations of KIAMB algorithm [6] were used. KIAMB was applied with Fisher's Z-test for continuous data (gene expression data) and G^2 test for discrete data (artificial simulated data), parameter $K = 0.8$, and three statistical thresholds $\alpha = 0.01$, $\alpha = 0.005$, and $\alpha = 0.001$ (denoted as KIAMB1, KIAMB2, KIAMB3, respectively). The first threshold was used by the inventors of the method in the paper that introduced it [6], while the latter two often lead to more parsimonious signatures without loss of predictivity based on prior experiments. A standard statistical threshold $\alpha = 0.05$ in most cases did not lead to termination of the algorithm, that is why it was not used in this work. To make experiments computationally tractable and robust to outlier runs of KIAMB, a 10 minute time limit was imposed for a single run of the algorithm.

Finally, an Iterative Removal method [7] was also applied. The implementation of this method used a signature extraction algorithm HITON-PC [8,9] since it typically yields more compact signatures with predictivity comparable or better to the other gene selection methods [8–11]. Statistical comparison tests to compare predictivity of the signatures [2,3] were also utilized.

References

1. Guyon I, Weston J, Barnhill S, Vapnik V (2002) Gene selection for cancer classification using support vector machines. Machine Learning 46: 389-422.

[1] Delong's test [2] was used to compare AUC point estimates in experiments with real gene expression data where the response variable had two categories. McNemar's test [3] was used to compare accuracies in experiments with simulated data where the response variable had more than two categories and AUC measure was not applicable.

2. DeLong ER, DeLong DM, Clarke-Pearson DL (1988) Comparing the areas under two or more correlated receiver operating characteristic curves: a nonparametric approach. Biometrics 44: 837-845.

3. Everitt, B. (1977) The analysis of contingency tables. London: Chapman and Hall.

4. Statnikov A, Aliferis CF, Tsamardinos I, Hardin D, Levy S (2005) A comprehensive evaluation of multicategory classification methods for microarray gene expression cancer diagnosis. Bioinformatics 21: 631-643.

5. Hollander, M. and Wolfe, D. (1999) Nonparametric statistical methods. New York, NY, USA: Wiley.

6. Peña J, Nilsson R, Björkegren J, Tegnér J (2007) Towards scalable and data efficient learning of Markov boundaries. International Journal of Approximate Reasoning 45: 211-232.

7. Natsoulis G, El GL, Lanckriet GR, Tolley AM, Leroy F, Dunlea S, Eynon BP, Pearson CI, Tugendreich S, Jarnagin K (2005) Classification of a large microarray data set: algorithm comparison and analysis of drug signatures. Genome Res 15: 724-736.

8. Aliferis CF, Statnikov A, Tsamardinos I, Mani S, Koutsoukos XD (2010) Local Causal and Markov Blanket Induction for Causal Discovery and Feature Selection for Classification. Part II: Analysis and Extensions. Journal of Machine Learning Research 11: 235-284.

9. Aliferis CF, Statnikov A, Tsamardinos I, Mani S, Koutsoukos XD (2010) Local Causal and Markov Blanket Induction for Causal Discovery and Feature Selection for Classification. Part I: Algorithms and Empirical Evaluation. Journal of Machine Learning Research 11: 171-234.

10. Aliferis CF, Statnikov A, Massion PP (2006) Pathway induction and high-fidelity simulation for molecular signature and biomarker discovery in lung cancer using microarray gene expression data. Proceedings of the 2006 American Physiological Society Conference "Physiological Genomics and Proteomics of Lung Disease" .

11. Aliferis CF, Statnikov A, Tsamardinos I, Kokkotou E, Massion PP (2006) Application and comparative evaluation of causal and non-causal feature selection algorithms for biomarker discovery in high-throughput biomedical datasets. Proceedings of the NIPS 2006 Workshop on Causality and Feature Selection .
