# Supplementary material for: Analysis and Computational Dissection of Molecular Signature Multiplicity
Source: PLoS Comput Biol. 2010 May 20;6(5):e1000790. doi: 10.1371/journal.pcbi.1000790 (PMC2873900; doi:10.1371/journal.pcbi.1000790)
Supplement: Text S5 — Data generation details. (0.13 MB PDF) [file pcbi.1000790.s013.pdf]

Data generation details

Generation of artificial simulated data

Using the principles from Figure 1 in the main manuscript, a discrete artificial network with 30 variables (29 genes and a phenotypic response variable T) was constructed such that all Markov boundaries were known. Figure below shows the network structure and specifies which genes contain the same information about T by the color of highlighting. For example, genes X_{12}, X_{13}, and X_{14} provide exactly the same information about T and thus are interchangeable for prediction of T. The parameterization of the network is provided in the table below. The network contains 72 Markov boundaries of T. Each of these Markov boundaries contains 5 genes: (i) X_{10}, (ii) X_5 or X_9, (iii) X_{12} or X_{13} or X_{14}, (iv) X_{19} or X_{20} or X_{21}, and (v) X_1 or X_2 or X_3 or X_{11}.

A discrete artificial network with 1,000 variables (999 genes and a phenotypic response variable T) was constructed by augmenting the network in the figure below with a total of 970 genes such that the resulting network has exactly the same 72 Markov boundaries. Out of 970 genes that were added to the prior network, 110 genes had a path to T and 860 genes did not.

Using the logic sampling method [1], we generated 3,750 observations from both networks. We used 750 observations for discovery of multiple signatures and the remaining 3,000 for validation of predictivity of T.

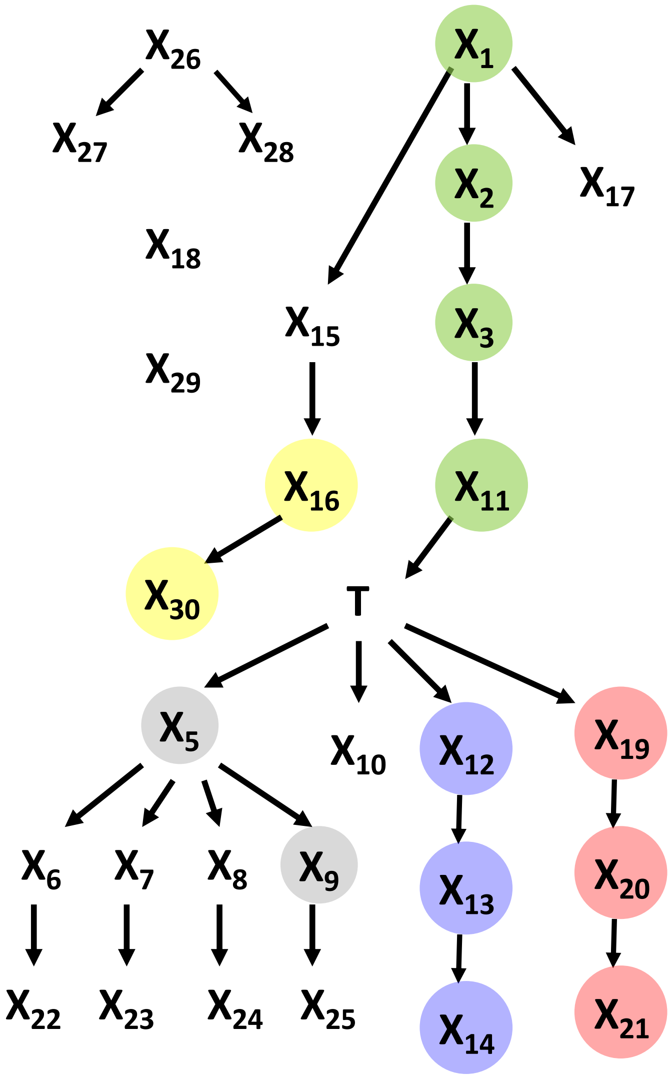

Figure: Graphical visualization of an artificial network with 30 variables (29 genes and phenotypic variable T). Genes that contain exactly the same information about T are highlighted with the same color, e.g. genes X_{12}, X_{13}, and X_{14} provide exactly the same information about T and thus are interchangeable for prediction of T.

X_1: $P(X_1=0) = 0.25$<br>$P(X_1=1) = 0.25$<br>$P(X_1=2) = 0.25$<br>$P(X_1=3) = 0.25$	X_6: $P(X_6=0\|X_5=0) = 0.6$<br>$P(X_6=1\|X_5=0) = 0.2$<br>$P(X_6=2\|X_5=0) = 0.2$<br>$P(X_6=0\|X_5=1) = 0.5$<br>$P(X_6=1\|X_5=1) = 0.25$<br>$P(X_6=2\|X_5=1) = 0.25$<br>$P(X_6=0\|X_5=2) = 0.8$<br>$P(X_6=1\|X_5=2) = 0.1$<br>$P(X_6=2\|X_5=2) = 0.1$	X_{11}: $P(X_{11}=0\|X_3=0) = 1.0$<br>$P(X_{11}=0\|X_3=1) = 1.0$<br>$P(X_{11}=1\|X_3=2) = 0.3$<br>$P(X_{11}=2\|X_3=2) = 0.7$<br>$P(X_{11}=3\|X_3=3) = 1.0$
X_2: $P(X_2=0\|X_1=0) = 0.8$<br>$P(X_2=1\|X_1=0) = 0.2$<br>$P(X_2=0\|X_1=1) = 0.1$<br>$P(X_2=1\|X_1=1) = 0.9$<br>$P(X_2=2\|X_1=2) = 1.0$<br>$P(X_2=3\|X_1=3) = 1.0$	X_7: $P(X_7=1\|X_5=0) = 0.5$<br>$P(X_7=2\|X_5=0) = 0.5$<br>$P(X_7=0\|X_5=1) = 0.8$<br>$P(X_7=1\|X_5=1) = 0.2$<br>$P(X_7=0\|X_5=2) = 0.2$<br>$P(X_7=1\|X_5=2) = 0.3$<br>$P(X_7=2\|X_5=2) = 0.5$	X_{12}: $P(X_{12}=0\|T=0) = 1.0$<br>$P(X_{12}=0\|T=1) = 1.0$<br>$P(X_{12}=0\|T=2) = 1.0$<br>$P(X_{12}=1\|T=3) = 0.5$<br>$P(X_{12}=2\|T=3) = 0.5$
X_3: $P(X_3=0\|X_2=0) = 0.3$<br>$P(X_3=1\|X_2=0) = 0.7$<br>$P(X_3=0\|X_2=1) = 0.8$<br>$P(X_3=1\|X_2=1) = 0.2$<br>$P(X_3=2\|X_2=2) = 1.0$<br>$P(X_3=3\|X_2=3) = 1.0$	X_8: $P(X_8=0\|X_5=0) = 0.9$<br>$P(X_8=1\|X_5=0) = 0.1$<br>$P(X_8=0\|X_5=1) = 0.7$<br>$P(X_8=1\|X_5=1) = 0.2$<br>$P(X_8=2\|X_5=1) = 0.1$<br>$P(X_8=0\|X_5=2) = 0.6$<br>$P(X_8=1\|X_5=2) = 0.3$<br>$P(X_8=2\|X_5=2) = 0.1$	X_{13}: $P(X_{13}=0\|X_{12}=0) = 1.0$<br>$P(X_{13}=1\|X_{12}=1) = 0.5$<br>$P(X_{13}=2\|X_{12}=1) = 0.5$<br>$P(X_{13}=1\|X_{12}=2) = 0.5$<br>$P(X_{13}=2\|X_{12}=2) = 0.5$
T: $P(T=0\|X_{11}=0) = 1.0$<br>$P(T=0\|X_{11}=1) = 1.0$<br>$P(T=0\|X_{11}=2) = 1.0$<br>$P(T=1\|X_{11}=3) = 0.3$<br>$P(T=2\|X_{11}=3) = 0.3$<br>$P(T=3\|X_{11}=3) = 0.4$	X_9: $P(X_9=1\|X_5=0) = 1.0$<br>$P(X_9=2\|X_5=1) = 1.0$<br>$P(X_9=0\|X_5=2) = 1.0$	X_{14}: $P(X_{14}=0\|X_{13}=0) = 1.0$<br>$P(X_{14}=1\|X_{13}=1) = 0.5$<br>$P(X_{14}=2\|X_{13}=1) = 0.5$<br>$P(X_{14}=1\|X_{13}=2) = 0.5$<br>$P(X_{14}=2\|X_{13}=2) = 0.5$
X_5: $P(X_5=1\|T=0) = 0.9$<br>$P(X_5=2\|T=0) = 0.1$<br>$P(X_5=0\|T=1) = 0.8$<br>$P(X_5=1\|T=1) = 0.1$<br>$P(X_5=2\|T=1) = 0.1$<br>$P(X_5=0\|T=2) = 0.1$<br>$P(X_5=1\|T=2) = 0.8$<br>$P(X_5=2\|T=2) = 0.1$<br>$P(X_5=0\|T=3) = 0.1$<br>$P(X_5=1\|T=3) = 0.1$<br>$P(X_5=2\|T=3) = 0.8$	X_{10}: $P(X_{10}=0\|T=0) = 0.1$<br>$P(X_{10}=1\|T=0) = 0.8$<br>$P(X_{10}=2\|T=0) = 0.1$<br>$P(X_{10}=1\|T=1) = 0.1$<br>$P(X_{10}=2\|T=1) = 0.9$<br>$P(X_{10}=0\|T=2) = 0.1$<br>$P(X_{10}=1\|T=2) = 0.8$<br>$P(X_{10}=2\|T=2) = 0.1$<br>$P(X_{10}=0\|T=3) = 0.2$<br>$P(X_{10}=1\|T=3) = 0.7$<br>$P(X_{10}=2\|T=3) = 0.1$	X_{15}: $P(X_{15}=0\|X_1=0) = 0.8$<br>$P(X_{15}=1\|X_1=0) = 0.1$<br>$P(X_{15}=2\|X_1=0) = 0.1$<br>$P(X_{15}=0\|X_1=1) = 0.1$<br>$P(X_{15}=1\|X_1=1) = 0.8$<br>$P(X_{15}=2\|X_1=1) = 0.1$<br>$P(X_{15}=0\|X_1=2) = 0.8$<br>$P(X_{15}=1\|X_1=2) = 0.1$<br>$P(X_{15}=2\|X_1=2) = 0.1$<br>$P(X_{15}=0\|X_1=3) = 0.1$<br>$P(X_{15}=1\|X_1=3) = 0.1$<br>$P(X_{15}=2\|X_1=3) = 0.8$

Table (continued on the next page): Parameterization of the network from the above figure. Only nonzero probabilities are shown in the table.

X_{16}: P(X_{16}=0|X_{15}=0) = 1.0<br>P(X_{16}=0|X_{15}=1) = 1.0<br>P(X_{16}=1|X_{15}=2) = 0.5<br>P(X_{16}=2|X_{15}=2) = 0.5	X_{21}: P(X_{21}=0|X_{20}=0) = 1.0<br>P(X_{21}=1|X_{20}=1) = 1.0<br>P(X_{21}=2|X_{20}=2) = 1.0	X_{26}: P(X_{26}=0) = 0.5<br>P(X_{26}=1) = 0.5
X_{17}: P(X_{17}=0|X_1=0) = 0.2<br>P(X_{17}=1|X_1=0) = 0.6<br>P(X_{17}=2|X_1=0) = 0.2<br>P(X_{17}=0|X_1=1) = 0.1<br>P(X_{17}=1|X_1=1) = 0.3<br>P(X_{17}=2|X_1=1) = 0.6<br>P(X_{17}=0|X_1=2) = 0.5<br>P(X_{17}=1|X_1=2) = 0.1<br>P(X_{17}=2|X_1=2) = 0.4<br>P(X_{17}=0|X_1=3) = 0.3<br>P(X_{17}=1|X_1=3) = 0.5<br>P(X_{17}=2|X_1=3) = 0.2	X_{22}: P(X_{22}=0|X_6=0) = 0.2<br>P(X_{22}=1|X_6=0) = 0.6<br>P(X_{22}=2|X_6=0) = 0.2<br>P(X_{22}=0|X_6=1) = 0.1<br>P(X_{22}=1|X_6=1) = 0.3<br>P(X_{22}=2|X_6=1) = 0.6<br>P(X_{22}=0|X_6=2) = 0.5<br>P(X_{22}=1|X_6=2) = 0.1<br>P(X_{22}=2|X_6=2) = 0.4	X_{27}: P(X_{27}=0|X_{26}=0) = 0.1<br>P(X_{27}=1|X_{26}=0) = 0.9<br>P(X_{27}=0|X_{26}=1) = 0.3<br>P(X_{27}=1|X_{26}=1) = 0.7
X_{18}: P(X_{18}=0) = 0.25<br>P(X_{18}=1) = 0.25<br>P(X_{18}=2) = 0.25<br>P(X_{18}=3) = 0.25	X_{23}: P(X_{23}=0|X_7=0) = 0.3<br>P(X_{23}=1|X_7=0) = 0.2<br>P(X_{23}=2|X_7=0) = 0.5<br>P(X_{23}=0|X_7=1) = 0.8<br>P(X_{23}=1|X_7=1) = 0.1<br>P(X_{23}=2|X_7=1) = 0.1<br>P(X_{23}=0|X_7=2) = 0.6<br>P(X_{23}=1|X_7=2) = 0.2<br>P(X_{23}=2|X_7=2) = 0.2	X_{28}: P(X_{28}=0|X_{26}=0) = 0.4<br>P(X_{28}=1|X_{26}=0) = 0.6<br>P(X_{28}=0|X_{26}=1) = 0.8<br>P(X_{28}=1|X_{26}=1) = 0.2
X_{19}: P(X_{19}=1|T=0) = 0.1<br>P(X_{19}=2|T=0) = 0.9<br>P(X_{19}=0|T=1) = 0.1<br>P(X_{19}=2|T=1) = 0.9<br>P(X_{19}=0|T=2) = 0.8<br>P(X_{19}=1|T=2) = 0.1<br>P(X_{19}=2|T=2) = 0.1<br>P(X_{19}=0|T=3) = 0.1<br>P(X_{19}=1|T=3) = 0.8<br>P(X_{19}=2|T=3) = 0.1	X_{24}: P(X_{24}=0|X_8=0) = 0.5<br>P(X_{24}=1|X_8=0) = 0.1<br>P(X_{24}=2|X_8=0) = 0.4<br>P(X_{24}=0|X_8=1) = 0.6<br>P(X_{24}=1|X_8=1) = 0.3<br>P(X_{24}=2|X_8=1) = 0.1<br>P(X_{24}=0|X_8=2) = 0.7<br>P(X_{24}=1|X_8=2) = 0.1<br>P(X_{24}=2|X_8=2) = 0.2	X_{29}: P(X_{29}=0) = 0.33<br>P(X_{29}=1) = 0.33<br>P(X_{29}=2) = 0.33
X_{20}: P(X_{20}=1|X_{19}=0) = 1.0<br>P(X_{20}=2|X_{19}=1) = 1.0<br>P(X_{20}=0|X_{19}=2) = 1.0	X_{25}: P(X_{25}=0|X_9=0) = 0.8<br>P(X_{25}=1|X_9=0) = 0.1<br>P(X_{25}=2|X_9=0) = 0.1<br>P(X_{25}=0|X_9=1) = 0.6<br>P(X_{25}=1|X_9=1) = 0.2<br>P(X_{25}=2|X_9=1) = 0.2<br>P(X_{25}=0|X_9=2) = 0.5<br>P(X_{25}=1|X_9=2) = 0.3<br>P(X_{25}=2|X_9=2) = 0.2	X_{30}: P(X_{30}=0|X_{16}=0) = 1.0<br>P(X_{30}=1|X_{16}=1) = 0.5<br>P(X_{30}=2|X_{16}=1) = 0.5<br>P(X_{30}=1|X_{16}=2) = 0.5<br>P(X_{30}=2|X_{16}=2) = 0.5

Table (continued from the previous page)

Generation of resimulated microarray gene expression data

The ability to produce realistic simulated data is a critical component of evaluating multiple signature identification algorithms in a systematic manner. In order to obtain large, realistic networks and data capturing the characteristics of human gene expression data, we applied a High-Fidelity Data Resimulation technique that generates synthetic data from a causal process that is induced from the real data and guarantees that the synthetic data is indistinguishable from the real data. Below we briefly outline the method and its application, more details can be found in [2].

The High-Fidelity Data Resimulation technique involves 6 steps[1]. *First*, a gene network is reverse-engineered from a real gene expression dataset. This step is performed by (a) obtaining an undirected graph by running HITON-PC algorithm for each gene and phenotype, (b) orienting the graph using greedy search-and-score learning with Bach's metric [3], and (c) learning densities of each gene and phenotype using SVM regression [4] and classification [5], respectively. *Second*, synthetic data is generated from the above network using logic sampling [1]. *Third*, a power-law relationship between genes and their connectivity is examined in the simulated network [6,7]. *Fourth*, a powerful classifier is applied to distinguish real from simulated data. The harder it is to perform this classification task, the better is the quality of resimulation. *Fifth*, Fisher's Z-test is used to ensure that statistical dependencies and independencies true in the real data are preserved in simulated data and vice-versa. *Sixth*, the existence of multiple maximally predictive and non-reducible signatures in simulated data is empirically demonstrated.

The above process was applied to 1,000 randomly selected variables (999 oligonucleotide gene probes and a phenotype variable) from the 12,600 gene probes in the Affymetrix U95A array lung cancer gene expression data of [8]. The phenotype variable denotes whether a subject has adenocarcinoma or squamous cell carcinoma. Once the network was reverse-engineered (step 1), a set of 30,000 samples was generated from this network (step 2). The synthetic network and data passed validation steps 3-6. More details are given in [2].

References

1. Russell, S. J. and Norvig, P. (2003) Artificial intelligence: a modern approach. Upper Saddle River, N.J: Prentice Hall/Pearson Education.

2. Aliferis CF, Statnikov A (2007) High-Fidelity Resimulation from High-Throughput Data. Technical Report DSL 07-03 .

3. Bach FR, Jordan MI (2003) Learning graphical models with Mercer kernels. Advances in Neural Information Processing Systems (NIPS) 15: 1009-1016.

4. Schölkopf, B., Burges, C. J. C., and Smola, A. J. (1999) Advances in kernel methods: support vector learning. Cambridge, Mass: MIT Press.

[1] Notice that steps 3-6 are used only for quality assurance purposes.

5. Vapnik, V. N. (1998) Statistical learning theory. New York: Wiley.

6. Barabasi AL, Bonabeau E (2003) Scale-free networks. Sci Am 288: 60-69.

7. Jeong H, Tombor B, Albert R, Oltvai ZN, Barabasi AL (2000) The large-scale organization of metabolic networks. Nature 407: 651-654.

8. Bhattacharjee A, Richards WG, Staunton J, Li C, Monti S, Vasa P, Ladd C, Beheshti J, Bueno R, Gillette M, Loda M, Weber G, Mark EJ, Lander ES, Wong W, Johnson BE, Golub TR, Sugarbaker DJ, Meyerson M (2001) Classification of human lung carcinomas by mRNA expression profiling reveals distinct adenocarcinoma subclasses. Proc Natl Acad Sci U S A 98: 13790-13795.
