# Supplementary material for: Analysis and Computational Dissection of Molecular Signature Multiplicity
Source: PLoS Comput Biol. 2010 May 20;6(5):e1000790. doi: 10.1371/journal.pcbi.1000790 (PMC2873900; doi:10.1371/journal.pcbi.1000790)
Supplement: Text S6 — Criteria for microarray gene expression dataset admissibility and protocol for quality assurance and processing. (0.04 MB PDF) [file pcbi.1000790.s014.pdf]

Criteria for microarray gene expression dataset admissibility and protocol for quality assurance and processing

Recall that discovery and validation datasets either originated from different laboratories or from different microarray platforms. We imposed the following criteria for dataset admissibility in the present work: same phenotype and same or very similar patient population in both datasets, both datasets produced by microarray platforms from Affymetrix, sample size in discovery dataset ≥ 100, and sample size in discovery dataset ≥ sample size in validation dataset. Once candidate pairs of discovery and validation datasets that satisfy the above criteria were obtained, we used the following quality assurance and processing procedure: (i) remove all patients/samples that are common between discovery and validation datasets (if applicable); (ii) for clinical outcome prediction tasks, remove censored patients/samples; (iii) if different microarray platforms are used, include only matching probes (obtained by using Affymetrix Array Comparison Spreadsheets: http://www.affymetrix.com/support/technical/comparison_spreadsheets.affx); (iv) ensure same or comparable normalization of both datasets; (v) verify presence of at least moderate predictive signal of the phenotype (>0.6 area under ROC curve) by using signature based on all genes, and finally (vi) ensure same or statistically indistinguishable performance of the signature based on all genes when trained and tested by holdout validation in the discovery dataset and when trained in the discovery dataset and tested in the validation dataset. The last step was used to ensure that the populations of patients/samples were comparable between the two datasets. To perform statistical testing in this step, we estimated 95% confidence intervals around each of the two point estimates[i] of area under ROC curve [1] and verified that at least one of these confidence intervals included a point estimate from another dataset.

References

1. DeLong ER, DeLong DM, Clarke-Pearson DL (1988) Comparing the areas under two or more correlated receiver operating characteristic curves: a nonparametric approach. Biometrics 44: 837-845.

[i] One point estimate is obtained when a classifier is trained and tested by holdout validation in the discovery dataset, and another one is obtained when a classifier is trained in the discovery dataset and tested in the validation dataset.
