# Supplementary material for: Analysis and Computational Dissection of Molecular Signature Multiplicity
Source: PLoS Comput Biol. 2010 May 20;6(5):e1000790. doi: 10.1371/journal.pcbi.1000790 (PMC2873900; doi:10.1371/journal.pcbi.1000790)
Supplement: Text S7 — The number of maximally predictive and non-redundant signatures is worst-case exponential to the number of variables. (0.08 MB PDF) [file pcbi.1000790.s015.pdf]

The number of maximally predictive and non-redundant signatures is worst-case exponential to the number of variables

Consider a simplified pathway structure and parameterization shown in the figure below. It involves n genes (X_1, X_2, ..., X_n) and a phenotypic response variable T. Genes X_i (i = 1,…,n) can be divided into m groups such that any two genes in a group contain exactly the same information about T. Since there are n/m genes in each group, the total number of Markov boundaries is $(n/m)^m$. Now assume that $m = kn$, where $k < 1$. Then the total number of Markov boundaries is $(1/k)^{kn}$. Since $1/k > 1$ and $kn = O(n)$, it follows that the number of Markov boundaries grows exponentially with the number of variables in this example.

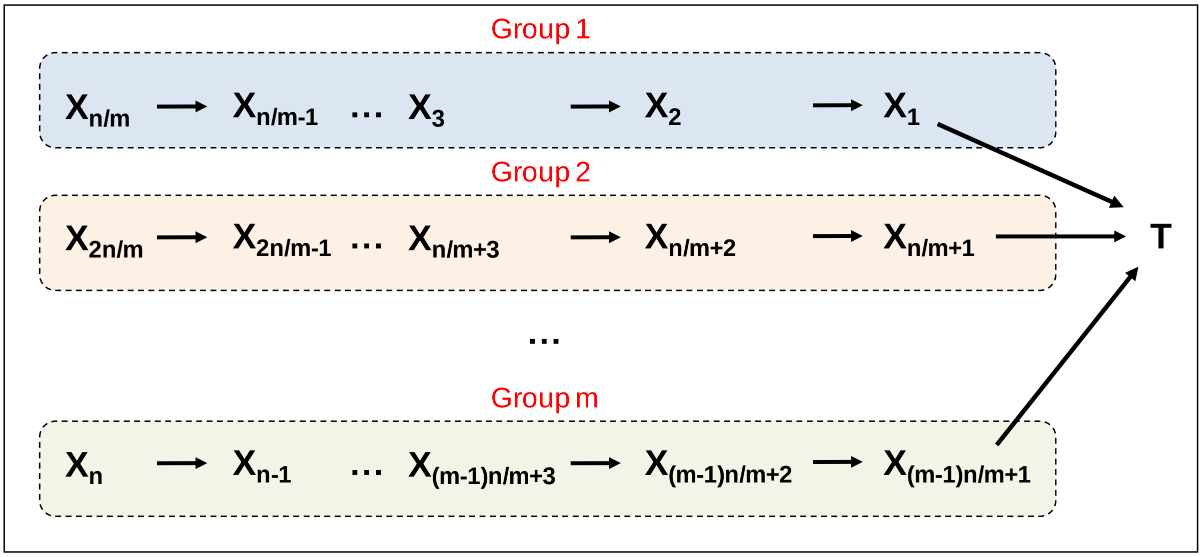

$P(T \mid X_1, X_{n/m+1},\ldots X_{(m-1)n/m+1})$	$(X_1 = 0, X_{n/m+1} = 0,\ldots X_{(m-1)n/m+1} = 0)$	$(X_1 = 0, X_{n/m+1} = 0,\ldots X_{(m-1)n/m+1} = 1)$	…	$(X_1 = 1, X_{n/m+1} = 1,\ldots X_{(m-1)n/m+1} = 1)$
$T = 0$	0.2	0.8		0.2
$T = 1$	0.8	0.2		0.8

For any pair of genes X_j and X_k belonging to the same group i:

$P(X_j \mid X_k)$	$X_k = 0$	$X_k = 1$
$X_j = 0$	1.0	0.0
$X_j = 1$	0.0	1.0

Figure: Example pathway structure with n gene variables (X_1, X_2, ..., X_n) and phenotypic response variable T. The structure is represented by a Bayesian network. The network parameterization is defined below the graph. All variables take values {0,1}.
