# Supplementary material for: Analysis and Computational Dissection of Molecular Signature Multiplicity
Source: PLoS Comput Biol. 2010 May 20;6(5):e1000790. doi: 10.1371/journal.pcbi.1000790 (PMC2873900; doi:10.1371/journal.pcbi.1000790)
Supplement: Text S8 — An example of signature multiplicity due to hidden variables. (0.02 MB PDF) [file pcbi.1000790.s016.pdf]

An example of signature multiplicity due to hidden variables

Consider a simplified pathway structure and parameterization shown in the figure below. It involves 3 genes (H, X_1, X_2) and a phenotypic response variable T. In the distribution with all variables observed, there is only one Markov boundary of T, which is $\{H\}$. Now consider that gene H is not observed in the data (i.e., it is hidden). Because H is not observed and genes X_1 and X_2 contain exactly the same information about T, two Markov boundaries $\{X_1\}$ and $\{X_2\}$ can be identified in this distribution. Notice that all these Markov boundaries have reproducible but suboptimal (relative to the original distribution with H observed) predictivity of the response variable T.

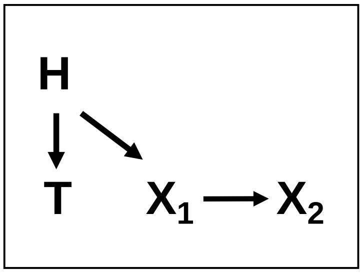

$P(T \mid H)$	$H = 0$	$H = 1$
$T = 0$	0.9	0.2
$T = 1$	0.1	0.8

$P(X_1 \mid H)$	$H = 0$	$H = 1$
$X_1 = 0$	0.9	0.1
$X_1 = 1$	0.1	0.9

$P(X_2 \mid X_1)$	$X_1 = 0$	$X_1 = 1$
$X_2 = 0$	1.0	0.0
$X_2 = 1$	0.0	1.0

Figure: Example pathway structure with 3 gene variables (H, X_1, X_2) and phenotypic response variable T. The structure is represented by a Bayesian network. The network parameterization is defined below the graph. All variables take values {0,1}.
