# Supplementary material for: Analysis and Computational Dissection of Molecular Signature Multiplicity
Source: PLoS Comput Biol. 2010 May 20;6(5):e1000790. doi: 10.1371/journal.pcbi.1000790 (PMC2873900; doi:10.1371/journal.pcbi.1000790)
Supplement: Text S9 — Preliminary experiments with the TIE* algorithm in mass-spectrometry proteomics data. (0.02 MB PDF) [file pcbi.1000790.s017.pdf]

**Preliminary experiments with the TIE* algorithm in mass-spectrometry proteomics data**

To demonstrate applicability of the TIE* algorithm to high-throughput data beyond gene expression microarrays, we describe here preliminary experiments with mass-spectrometry proteomics data from [1]. The dataset contains 216 samples: 95 from healthy controls and 121 from ovarian cancer patients. The phenotypic response variable is case versus control status. Before running TIE*, we applied baseline subtraction with peak detection [2] and peak alignment [3]. This resulted in a dataset with 2,190 peaks.

In order to ensure unbiased estimation of AUC of molecular signatures output by TIE*, we used hold-out cross-validation. Specifically, 75% of samples were used for running the TIE* algorithm to find genes that participate in multiple signatures and for training support vector machine classifiers. The remaining 25% of samples were used for estimation of AUC of signatures. We emphasize that neither TIE* nor SVM had access to these testing samples. Since hold-out cross-validation may have high variance in general, we applied the above protocol 10 times with random splits into training and testing sets. All algorithms were applied exactly with the same parameters as in gene expression experiments from the main manuscript. The results are provided in the table below:

| Split into training and testing sets | Number of signatures found by TIE* | AUC of all signatures identified by TIE* | |
|---|---|---|---|
| | | Mean | 95% Interval |
| #1 | 198 | 0.977 | [0.934, 0.998] |
| #2 | 258 | 0.977 | [0.939, 0.993] |
| #3 | 116 | 0.961 | [0.939, 0.986] |
| #4 | 189 | 0.948 | [0.923, 0.975] |
| #5 | 267 | 0.967 | [0.925, 0.997] |
| #6 | 232 | 0.956 | [0.925, 0.987] |
| #7 | 286 | 0.971 | [0.940, 0.994] |
| #8 | 232 | 0.969 | [0.940, 0.989] |
| #9 | 181 | 0.969 | [0.940, 0.994] |
| #10 | 221 | 0.961 | [0.929, 0.991] |

As can be seen, TIE* identified hundreds of ovarian cancer signatures with almost perfect AUC estimates (= 0.95-0.98). Each signature contains only 5 peaks on average. It is also worth noting that each run of the TIE* algorithm completed within ~1-2 minutes on a workstation with 2.4 GHz Intel Xeon CPU and 12 GB of RAM.

**References**


1. Conrads TP, Fusaro VA, Ross S, Johann D, Rajapakse V, Hitt BA, Steinberg SM, Kohn EC, Fishman DA, Whitely G, Barrett JC, Liotta LA, Petricoin EF, III, Veenstra TD (2004) High-resolution serum proteomic features for ovarian cancer detection. Endocr Relat Cancer 11: 163-178.

2. Coombes KR, Fritsche HA, Jr., Clarke C, Chen JN, Baggerly KA, Morris JS, Xiao LC, Hung MC, Kuerer HM (2003) Quality control and peak finding for proteomics data collected from nipple aspirate fluid by surface-enhanced laser desorption and ionization. Clin Chem 49: 1615-1623.

3. Yasui Y, McLerran D, Adam BL, Winget M, Thornquist M, Feng Z (2003) An automated peak identification/calibration procedure for high-dimensional protein measures from mass spectrometers. Journal of Biomedicine and Biotechnology 4: 242-248.
